# The ESX-1 Substrate PPE68 Has a Key Function in ESX-1-Mediated Secretion in Mycobacterium marinum

**DOI:** 10.1128/mbio.02819-22

**Published:** 2022-11-21

**Authors:** Merel P. M. Damen, Aniek S. Meijers, Esther M. Keizer, Sander R. Piersma, Connie R. Jiménez, Coenraad P. Kuijl, Wilbert Bitter, Edith N. G. Houben

**Affiliations:** a Section of Molecular Microbiology, Amsterdam Institute of Molecular and Life Sciences, Vrije Universiteit Amsterdam, Amsterdam, The Netherlands; b Department of Medical Microbiology and Infection Control, Amsterdam UMC, VU Medical Center, Amsterdam, The Netherlands; c Department of Medical Oncology, OncoProteomics Laboratory, Cancer Center Amsterdam, Amsterdam UMC, VU Medical Center, Amsterdam, The Netherlands; Weill Cornell Medical College

**Keywords:** ESX-1, EsxA, *Mycobacterium*, PPE, chaperones, protein transport, protein-protein interactions, tuberculosis, type VII secretion

## Abstract

Mycobacteria use specialized type VII secretion systems (T7SSs) to secrete proteins across their diderm cell envelope. One of the T7SS subtypes, named ESX-1, is a major virulence determinant in pathogenic species such as Mycobacterium tuberculosis and the fish pathogen Mycobacterium marinum. ESX-1 secretes a variety of substrates, called Esx, PE, PPE, and Esp proteins, at least some of which are folded heterodimers. Investigation into the functions of these substrates is problematic, because of the intricate network of codependent secretion between several ESX-1 substrates. Here, we describe the ESX-1 substrate PPE68 as essential for secretion of the highly immunogenic substrates EsxA and EspE via the ESX-1 system in M. marinum. While secreted PPE68 is processed on the cell surface, the majority of cell-associated PPE68 of M. marinum and M. tuberculosis is present in a cytosolic complex with its PE partner and the EspG_1_ chaperone. Interfering with the binding of EspG_1_ to PPE68 blocked its export and the secretion of EsxA and EspE. In contrast, *esxA* was not required for the secretion of PPE68, revealing a hierarchy in codependent secretion. Remarkably, the final 10 residues of PPE68, a negatively charged domain, seem essential for EspE secretion, but not for the secretion of EsxA and of PPE68 itself. This indicates that distinctive domains of PPE68 are involved in secretion of the different ESX-1 substrates. Based on these findings, we propose a mechanistic model for the central role of PPE68 in ESX-1-mediated secretion and substrate codependence.

## INTRODUCTION

The genus Mycobacterium belongs to the phylum *Actinobacteria* and includes the major human pathogens Mycobacterium tuberculosis and Mycobacterium leprae. Mycobacteria have a distinctive cell envelope that is shared with other bacteria within the order *Corynebacteriales*. The unique feature of their cell envelope is the long-chained fatty acids, called mycolic acids, that assemble into a second hydrophobic bilayer, also known as the mycobacterial outer membrane (1, 2). Mycobacteria have acquired specialized secretion systems, called type VII secretion systems (T7SSs), to export proteins across this multilayered cell envelope. There are five paralogous T7SSs present in M. tuberculosis, called ESX-1 to ESX-5. The ESX-1 system is considered a major virulence factor of M. tuberculosis and the fish pathogen Mycobacterium marinum through its essential role in phagosomal rupture and subsequent translocation of the pathogen to the host cytosol (3–6). As a consequence, the absence of the *esx-1* region results in severe attenuation of pathogenic mycobacteria, which is further illustrated by the observation that a large deletion within the same region (RD1) is the prime cause of the attenuation of the live vaccine strain Mycobacterium bovis BCG (7–9).

The ESX-1 system secretes over a dozen different proteins, which all belong to the EsxAB clan (Pfam CL0352) and can be categorized into four distinctive protein families, i.e., Esx/WxG100, PE, PPE, and Esp. The best-studied and highly immunogenic ESX-1 substrates EsxA and EsxB belong to the Esx/WxG100 protein family, which are characterized by a WxG protein motif and generally are 100 amino acids long. PE35 and PPE68 are encoded within the same operon as *esxA* and *esxB* and belong to PE and PPE protein families that share homologous N-terminal domains of approximately 110 amino acids and 180 amino acids, respectively, with conserved proline-glutamic acid (PE) and proline-proline-glutamic acid (PPE) motifs (10). While each T7SS secretes its own Esx proteins and often also PE and PPE substrates, the Esp proteins seem to be exclusively secreted by the ESX-1 system.

An intriguing feature of the mycobacterial T7SS substrates is that these proteins are secreted as folded heterodimers that are formed in the cytosol (11–14). While these heterodimers are formed by either Esx substrates or a PE protein with a specific PPE protein, available crystal structures reveal highly conserved features. Each complex forms a four-helix bundle, composed of two helix-turn-helix structures in an antiparallel orientation, with a conserved secretion motif (YxxxD/E) extending from the double-helix of one partner protein (15). In addition to the helix-turn-helix, the N-terminal domain of PPE proteins contains an extra region that is relatively hydrophobic and extends from the PE-PPE interface (16). This so-called helical tip is recognized by a cytosolic chaperone, called EspG, in an ESX system-specific manner, recognition of which is required for secretion of the PE-PPE pair (12, 13, 17). In addition to the conserved dimeric structure formed by the N-terminal PE and PPE domains, which is thought to be involved in substrate recognition and secretion of the heterodimers, many PE and PPE proteins have highly variable C-terminal domains that could potentially execute specific functions (18–20). Only limited structural information is available for the Esp substrates, although structure predictions indicate that most of these substrates form similar heterodimers as PE and PPE proteins (21–23).

EsxA has been indicated to play a central role in ESX-1-mediated virulence, including the perturbation of phagosomal membranes (3, 4, 6, 24–26). However, while *in vitro* biochemical analyses suggest that EsxA has membranolytic activity (25, 27–31), later reports have ascribed at least some of these observations to detergent contaminations during the purification of EsxA (30, 32). Another complicating factor is that mutations in *esxA* abolish the secretion of other ESX-1 substrates, such as EspA, EspF, EspJ, EspK, and EspB in M. tuberculosis and/or M. marinum (33–35). In numerous cases, the codependence for secretion is mutual, as the deletion of specific *esp* genes also results in abolished EsxA secretion (34, 36–39). This complicates the analysis and interpretation of the role of individual ESX-1 substrates in virulence.

Codependent secretion has also been observed for substrates of other ESX systems (40–42). The underlying reason for this phenomenon, however, remains unclear. Previously, we provided the first mechanistic insight into this process by the successful redirection of PE35_1/PPE68_1, the M. marinum paralogs of the *esx-1* locus-encoded PE35/PPE68, from the ESX-1 to the ESX-5 system (43, 44). Surprisingly, the redirection of these two ESX-1 substrates also resulted in redirection of EsxA_1. This finding suggested a central role of the PE-PPE-EspG complex in the secretion of Esx substrates. Interestingly, deletion of *espG_1_* results in a general secretion defect not only of respective PE/PPE and Esx substrates in M. marinum but also of Esp proteins (24, 33, 35, 39, 45, 46), indicating a central role for PE/PPE substrates in the secretion of all known ESX substrate classes. Here, we investigated the role of PPE68 in ESX-1-mediated secretion of EsxA and EspE in M. marinum, and we report that this PPE protein has a central function in the secretion of these two ESX-1 substrates.

## RESULTS

### The *pe35/ppe68* pair is necessary for secretion of ESX-1 substrates EsxA and EspE and for ESX-1-mediated hemolysis.

**FIG 1:**
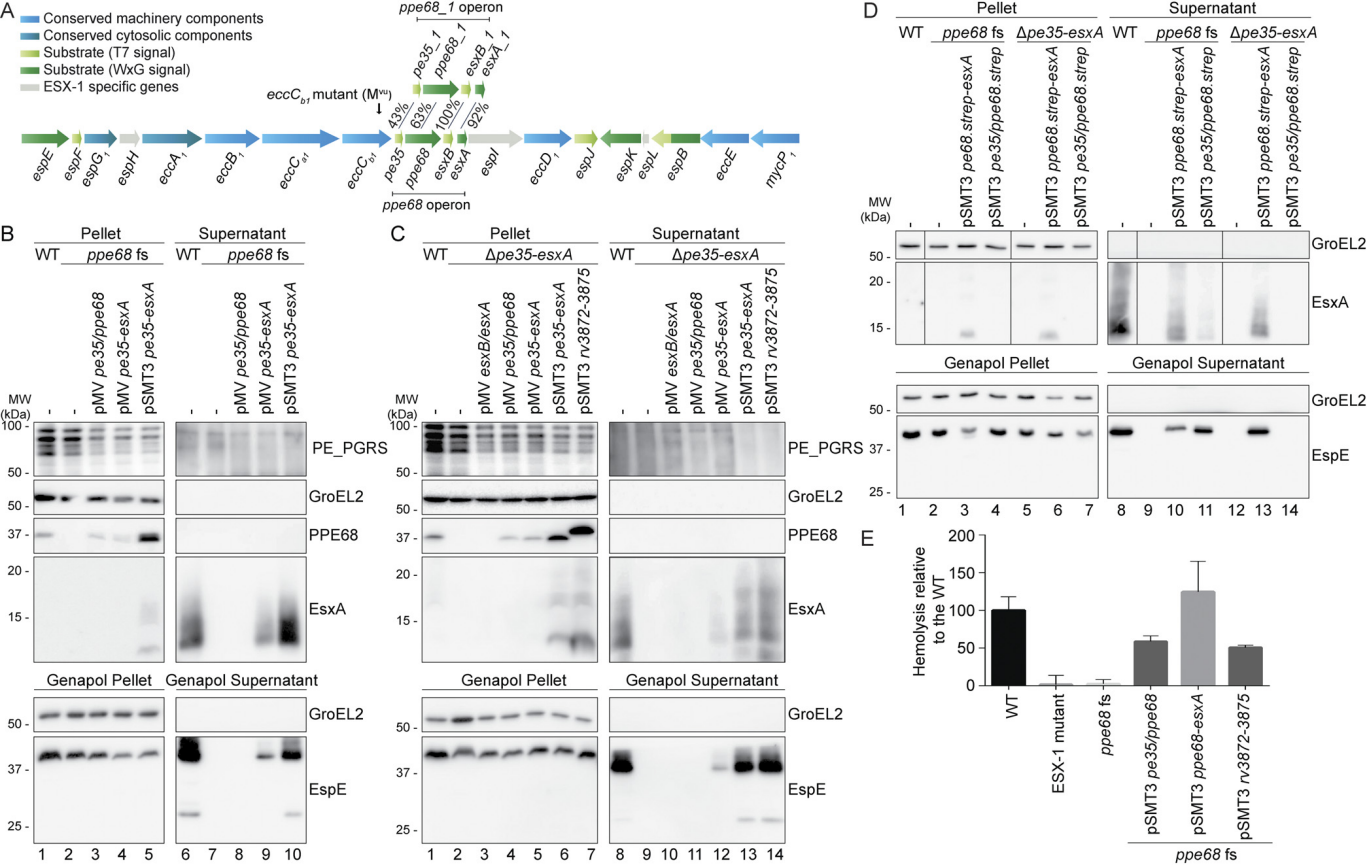
*pe35/ppe68* is involved in the secretion of ESX-1 substrates EsxA and EspE, and is required for ESX-1 mediated hemolysis. A) Schematic overview of the esx-1 gene cluster and its paralogous region. Genes are color-coded to their subcellular localization (key color). Notably, the pe gene upstream of *ppe68_1* was originally named *pe35*, while the paralogous gene upstream of *ppe68* in the esx-1 locus was named *mmar_5447*. In this study, we renamed these two genes *pe35_1* and *pe35*, respectively. B, C, D) SDS-PAGE and immunostaining of the cell pellet, culture supernatant, Genapol pellet and Genapol supernatant fractions of M. marinum M wild type (WT) and the *ppe68* fs mutant without and with various complementation constructs (B, D) or WT and the *ppe68* operon mutant (Δpe35-*esxA*) with and without various complementation constructs (C, D). Genes were expressed from an integrative pMV vector or a multicopy pSMT3 vector under control of the hsp60 promotor, and for D) with a Strep-tag on the C-terminus of PPE68. In B and D) the WT, *ppe68* fs mutant and the ppe68 fs mutant containing a pSMT3 vector also contained a pMV TdTomato plasmid. Proteins were visualized with anti-EsxA, anti-EspE, and anti-*PPE68* antibodies (ESX-1 substrates). A processed band of 25 kDa was detected by the anti-EspE antibody in the Genapol supernatant fraction of the WT, which has been reported before (46). As a loading and lysis control, blots were incubated with antibodies directed against the cytosolic GroEL2 protein and secreted PE_PGRS proteins (ESX-5 substrates). In all blots, equivalent OD units were loaded: 0.2 OD for pellet and Genapol pellet and 0.4 OD for supernatant and Genapol supernatant fractions (B, C) or 0.2 OD for supernatant fractions and 2 OD for Genapol supernatant fractions (D). Data shown are representative of three independent experiments. E) Hemoglobin release after incubation of sheep red blood cells with *M. marinum* WT, an ESX-1 mutant (*eccC_b1_* mutant; M^VU^), the *ppe68* fs mutant, and the *ppe68* fs mutant complemented with either the entire *ppe68* operon of *M. marinum*, with only *pe35/ppe68*, or with the *ppe68* operon of *M. tuberculosis* (rv3872-rv3875). Hemoglobin release was quantified by determining the OD450 of the medium after incubation. Data shown are from two independent experiments including 3 technical replicates each.

To study the effect of PPE68 on protein secretion, we created a frameshift mutation in M. marinum M to minimize polar effects, using the efficient genome editing tool CRISPR1-Cas9 (47). The generated *ppe68* frameshift (fs) mutant that we selected contained a 1-bp insertion at position 181, which resulted in an early stop codon after amino acid 202 (see Fig. S1A in the supplemental material). As expected, PPE68 could not be detected in the pellet or supernatant fraction of this mutant (Fig. 1B, lanes 2 and 7). In the wild-type (WT) strain, we could identify PPE68 in the pellet fraction but not in the supernatant (Fig. 1B, lanes 1 and 6), which is in line with the observations in M. tuberculosis (48). The *ppe68* mutant showed a loss of EsxA in the supernatant fraction, while no accumulation of EsxA in the pellet fraction was observed (Fig. 1B, lanes 2 and 7). Similarly, no EsxA accumulation was observed in the cell pellet of an *eccCb_1_* core complex mutant (Fig. S1C, lane 2), an observation that has previously been linked to downregulated expression of *esxA* in the presence of an inactive ESX-1 secretion system by a WhiB6-controlled negative feedback mechanism (49, 50). In agreement, we observed that mRNA levels of *esxA* in the *ppe68* fs mutant was reduced to a comparable level as in the *eccCb_1_* mutant (Fig. S1D). Next, we analyzed the secretion of ESX-1 substrate EspE, which is an abundant cell surface protein of M. marinum and can be extracted by the mild detergent Genapol X-080 (51, 52). The *ppe68* fs mutation prevented the secretion of EspE to the cell surface, while the intracellular levels of EspE in the mutant and the WT were comparable (Fig. 1B, lanes 2 and 7). Complementation of the *ppe68* fs mutant with the *pe35/ppe68* pair, expressed from an integrative pMV vector, failed to restore detectable EspE and EsxA secretion (Fig. 1B, lane 8), while the introduction of the complete *ppe68* operon, encompassing *pe35*, *ppe68*, *esxB*, and *esxA*, using either the pMV or multicopy pSMT3 vector, restored EspE and EsxA secretion (Fig. 1B, lanes 9 and 10). Importantly, expression of *pe35*/*ppe68* from the pSMT3 vector, with a Strep tag at the C terminus of PPE68 for detection purposes, also fully restored EspE secretion, while EsxA was secreted at lower levels than in the WT (Fig. 1D, lane 11). Notably, a similarly reduced amount of *esxA* transcripts was observed in this latter complementation strain as in the *ppe68* fs mutant (Fig. S1D), showing that these mRNA levels were sufficient for EsxA secretion and therefore that downregulated transcription was not the main cause for the absence of EsxA in the culture supernatant of the mutant.

10.1128/mbio.02819-22.1 FIG S1 Generation and confirmation of M. marinum frameshift mutants, including phenotypic analysis of the *ppe68* fs mutant. (A, B) Each panel shows the sgRNA binding region within the gene, i.e. in *ppe68* (A) and *mmar_2894* (B), with a panel underneath showing the sequencing results that were obtained after PCR. (C) SDS-PAGE and immunostaining of the cell pellet fraction of M. marinum WT, the *eccC_b1_* mutant (M^VU^ – ESX-1 mutant), the *ppe68* fs mutant and the *mmar_2894* fs mutant. The *ppe68* operon mutant (Δ*pe35-esxA*) with and without the *ppe68* operon expressed from a multicopy pSMT3 vector under control of the *hsp60* promotor served as the positive and negative control for EsxA production, respectively. The *ppe68* fs mutant and *mmar_2894* fs mutant also contained a pMV TdTomato plasmid. Proteins were visualized with an anti-EsxA antibody, and as a loading control, blots were incubated with antibodies directed against the cytosolic GroEL2 protein. An amount of 0.2 OD units was loaded for the pellet fraction. Data are representative of two independent experiments. (D) Relative *esxA* mRNA levels of an *eccC_b1_* mutant (M^VU^, ESX-1 mutant), the *ppe68* fs mutant, and the *ppe68* fs mutant complemented with *pe35/ppe68* expressed from the pMST3 vector, compared to the WT. Cycle threshold (*C_T_*) values were normalized for *C_T_* values of the household gene *sigA* and compared to the other *C_T_* values. Data shown are from two independent experiments including 3 technical replicates each. Download FIG S1, TIF file, 1.5 MB. Copyright © 2022 Damen et al. 2022 Damen et al. https://creativecommons.org/licenses/by/4.0/ This content is distributed under the terms of the Creative Commons Attribution 4.0 International license.

To investigate the individual roles of *pe35/ppe68* and *esxB*/*esxA* in ESX-1-mediated secretion, we created an M. marinum knockout strain that contained a deletion of the complete *pe35-ppe68-esxA-esxB* operon (Fig. 1A). Again, no EspE was detected on the cell surface of the *ppe68* operon mutant, while the intracellular EspE levels were comparable to those in the WT (Fig. 1C, lanes 2 and 9). Introduction of either *esxB/esxA* or *pe35/ppe68* on the integrative pMV vector, or for *pe35/ppe68*, also on the pSMT3 plasmid, failed to restore EspE and EsxA secretion (Fig. 1C, lanes 10 and 11, and Fig. 1D, lane 14). EspE and EsxA secretion was only restored when the entire *ppe68* operon was present on the introduced pMV or pSMT3 vector (Fig. 1C, lanes 12 and 13). Expression of the entire *ppe68* operon from the pSMT3 vector resulted in secretion levels similar to those in the WT. In addition, the *ppe68* operon of M. tuberculosis was also able to restore EspE and EsxA secretion by the *ppe68* operon mutant, showing that the function of the *ppe68* operon in ESX-1 secretion was conserved (Fig. 1C, lane 14).

Next, we investigated the impact of the *ppe68* fs mutation on ESX-1-mediated membrane lysis as an important readout for ESX-1-mediated virulence mechanisms (24). As expected, while WT M. marinum lysed sheep red bloods cells efficiently, the *eccC_b1_* mutant showed no effect above background levels (Fig. 1E). Also, the *ppe68* fs mutant completely lost its hemolytic capabilities, which could be fully complemented by the introduction of the *ppe68* operon. Introduction of the *pe35/ppe68* pair on a pSMT3 plasmid and of the *ppe68* operon of M. tuberculosis partially restored hemolysis (Fig. 1E). In conclusion, *ppe68* is involved in the hemolytic activity of M. marinum and the secretion of EsxA and EspE, a role that is conserved between the M. marinum and M. tuberculosis protein.

### The EspG_1_-binding domain of PPE68 is important for its central role in ESX-1 secretion.

**FIG 2:**
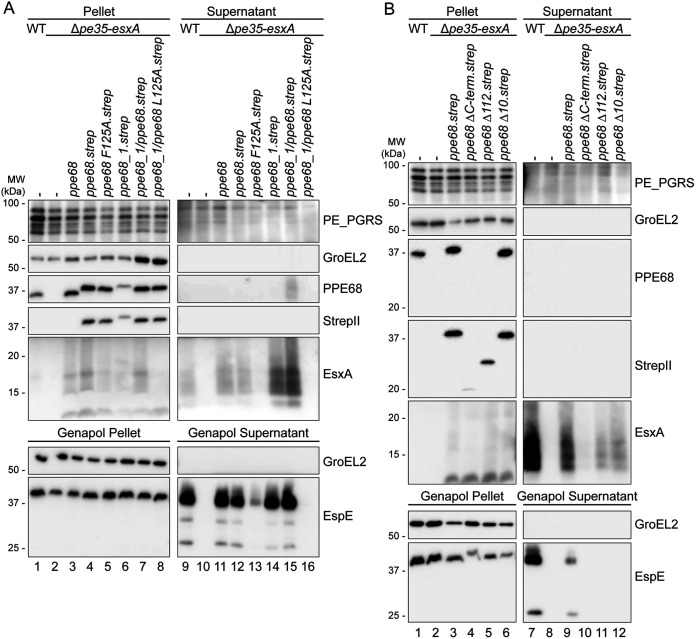
Conserved features of PPE68 are important for its central role in ESX-1 secretion. (A) Role of the EspG_1_-binding domain of PPE68. SDS-PAGE and immunostaining of the cell pellet, culture supernatant, Genapol pellet, and Genapol supernatant fractions of M. marinum WT, the *ppe68* operon mutant (Δ*pe35-esxA*), and the *ppe68* operon mutant complemented with either the *ppe68* operon, the *ppe68_1* operon, or a hybrid operon containing *pe35_1*, the N-terminal domain of *ppe68_1*, or the C-terminal domain of *ppe68*, *esxB*, and *esxA*. Single amino acid substitutions were made in the helical tip of PPE68 and the PPE68_1/PPE68 hybrid to investigate the role of the EspG_1_-binding domain. (B) The role of the C-terminal domain of PPE68. SDS-PAGE and immunostaining of the cell pellet, culture supernatant, Genapol pellet, and Genapol supernatant fractions of M. marinum WT, the *ppe68* operon mutant (Δ*pe35-esxA*), and the *ppe68* operon mutant complemented with either the WT or *ppe68* operon variants, encoding PPE68 lacking either the entire (PPE68ΔC-term) or partial, i.e., the final 112 (PPE68Δ112) or 10 (PPE68Δ10) amino acids, of the C-terminal domain. In both panels A and B, the operons were expressed from a multicopy pSMT3 vector under control by the *hsp60* promoter and with a Strep tag on the C terminus of PPE68 and its variants. Proteins were visualized with anti-EsxA, anti-EspE, anti-StrepII (PPE68), and anti-PPE68 antibodies (ESX-1 substrates). A processed band of 25 kDa was detected by the anti-EspE antibody in the Genapol supernatant fraction of the WT, which has been reported before (Phan et al., 2018 [46]). As a loading and lysis control, blots were incubated with antibodies directed against the cytosolic GroEL2 protein and secreted PE_PGRS proteins (ESX-5 substrates). In all blots, equivalent OD units were loaded: 0.2 OD for pellet and Genapol pellet and 0.4 OD for supernatant and Genapol supernatant fractions. Data shown in each panel are representative of three independent experiments.

Previously, we reported that the EspG_1_-binding domain of PPE68_1 (MMAR_0186) is essential for secretion via the ESX-1 system (44). Specifically, a single amino acid substitution at position 125, located within this domain, abrogated PPE68_1 secretion to the culture supernatant without affecting its production. Similarly, the conserved amino acid in an ESX-5-dependent PPE protein (L125) was essential for EspG_5_ binding and its secretion (13). To investigate the role of EspG_1_ binding to PPE68, an equivalent F to A amino acid change at position 125 (F125A) was introduced in the *ppe68* operon on the pSMT3 vector, again with a Strep tag to the C terminus of PPE68 for detection purposes. Immunoblot analysis showed that PPE68.strep was properly produced and, as expected, ran slightly higher on an SDS-PAGE gel (Fig. 2A, lane 4). In addition, the level of EsxA and EspE secretion by the complemented *ppe68* operon mutant was not affected by the Strep tag (Fig. 2A, lane 12). While the F125A substitution did not affect the stability of this PPE68 variant (Fig. 2A, lane 5), it could not restore WT levels of EsxA secretion and showed severely reduced EspE secretion in the *ppe68* operon mutant (Fig. 2A, lane 13). Notably, unlike for the *ppe68* fs mutant, EsxA could be detected in the bacterial pellet, showing that this protein is stably produced from the PPE68 F125A operon plasmid. This result indicated that recognition of PPE68 by EspG_1_ is indeed important for its role in ESX-1 secretion.

As we were unable to directly assess the secretion of PPE68, but previously could observe secretion of PPE68_1 (43, 44), we further investigated the paralogous *ppe68_1* operon, consisting of *pe35_1*, *ppe68_1*, *esxB_1*, and *esxA_1* (Fig. 1A). While *esxB_1* and *esxA_1* were almost identical to their paralogs in the *esx-1* gene cluster, *pe35_1* and *ppe68_1* showed less similarity, particularly for the C-terminal domains of the PPE proteins (Fig. 1A and Fig. S2). Notably, deletion of this four-gene paralogous region did not affect EspE secretion nor the hemolytic activity of M. marinum, showing that these endogenous genes are dispensable for these specific ESX-1-mediated processes (Fig. S3). To assess the importance of the C-terminal domain of the PPE68 paralogs in ESX-1 secretion, we constructed a pSMT3 plasmid containing the *ppe68_1* operon (*mmar_0185-88*) and a *ppe68*/*ppe68_1* hybrid operon construct, containing *pe35_1*, the N-terminal domain of *ppe68_1* (PPE domain, residues 1 to 175) (Fig. S2), the C-terminal domain of *ppe68* (encoding residues 176 to 367), *esxA*, and *esxB*. Again, a Strep tag was introduced to the C terminus of the PPE variants for detection purposes. Surprisingly, both the *ppe68_1* operon and the hybrid operon reestablished EsxA and EspE secretion in the *ppe68* operon mutant (Fig. 2A, lanes 14 and 15), while the *ppe68_1* operon was also able to restore hemolytic activity in the *ppe68* fs mutant (Fig. S3B). These results showed that the paralogous but divergent PPE68_1 is functionally similar to the PPE68 with regard to their role ESX-1 secretion. However, the fact that deleting the paralogous PPE68_1 region did not impact ESX-1 secretion indicated that this region was not expressed under the conditions used. Notably, while some small amounts of hemagglutinin (HA)- or FLAG-tagged PPE68_1 could previously be detected in culture supernatants of M. marinum M (43, 44), PPE68_1 with a Strep tag could not clearly be detected in this fraction (Fig. 2A, lane 14). In contrast, the PPE68_1/PPE68 hybrid could readily be observed in the culture supernatant using the anti-PPE68 antibody (Fig. 2A, lane 15). The anti-StrepII antibody failed to visualize this protein in this fraction, likely due to C-terminal processing of the PPE68_1/PPE68 hybrid upon secretion (see below). Most importantly, the introduction of an L125A mutation in the EspG_1_-binding domain of the PPE68_1/PPE68 hybrid completely blocked its ability to complement secretion of EspE and EsxA in the *ppe68* operon mutant and also abrogated the secretion of the PPE68_1/PPE68 hybrid itself (Fig. 2A, lane 16). These combined results show that the amino acid in position 125, located within the helical tip of the EspG_1_-binding domain, is essential for the export of the PPE68/PPE68_1 hybrid, which in turn is necessary for the secretion EspE and EsxA.

10.1128/mbio.02819-22.2 FIG S2 Protein sequence alignment of PPE68 homologs of M. marinum and M. tuberculosis, including the secondary structure prediction of M. tuberculosis PPE68. The secondary structure prediction was produced by Alphafold2. The blue square indicates the residue at position 125 involved in EspG_1_ binding. Underlined sequences indicate the C-terminal truncations according to the color key. Rv3873, PPE68 of M. tuberculosis; MMAR_5448, PPE68 of M. marinum; MMAR_0186, PPE68_1 of M. marinum. Download FIG S2, TIF file, 1.6 MB. Copyright © 2022 Damen et al. 2022 Damen et al. https://creativecommons.org/licenses/by/4.0/ This content is distributed under the terms of the Creative Commons Attribution 4.0 International license.

10.1128/mbio.02819-22.3 FIG S3 Deletion of the paralogous *ppe68_1* operon does not affect EspE secretion or hemolytic activity of M. marinum. (A) SDS-PAGE and immunostaining were performed on the cell pellet, culture supernatant, Genapol pellet, and Genapol supernatant fractions of M. marinum WT, an ESX-1 mutant (*eccC_b1_* mutant; M^VU^), the *ppe68* operon mutant (Δ*pe35-esxA*), and the *ppe68_1* operon mutant (Δ*pe35_1-esxA_1*). Proteins were visualized with anti-EspE and anti-PPE68 (ESX-1 substrates) antibodies. A processed band of 25 kDa was detected by the anti-EspE antibody in the Genapol supernatant fraction of the WT, which has been reported before (Phan et al., 2018 [46]). As a loading and lysis control, blots were incubated with antibodies directed against the cytosolic GroEL2 protein. In all blots, equivalent OD units were loaded: 0.2 OD for pellet and Genapol pellet and 0.4 OD for supernatant and Genapol supernatant fractions. Data shown are representative of two independent experiments. (B) Hemoglobin release after incubation of the same strains as in panel A and the *ppe68* fs mutant expressing the *ppe68_1* operon with sheep red blood cells. Hemoglobin release was quantified by determining the OD_450_ of the medium after incubation. Data shown are from two independent experiments including 3 technical replicates each. Download FIG S3, TIF file, 0.8 MB. Copyright © 2022 Damen et al. 2022 Damen et al. https://creativecommons.org/licenses/by/4.0/ This content is distributed under the terms of the Creative Commons Attribution 4.0 International license.

### The C-terminal domain of PPE68 is necessary for ESX-1-mediated secretion.

While the C-terminal domains of PPE68_1 and PPE68 are relatively dissimilar, they possess a well-conserved stretch of 15 amino acids consisting of an unusual high number of negatively charged residues at their C termini. To investigate the role of the C-terminal domain of PPE68 in more detail, several C-terminal truncations were made, namely, PPE68ΔC (residues 1 to 174), PPE68Δ112 (residues 1 to 256), and PPE68Δ10 (residues 1 to 357) (Fig. S2), including a C-terminal Strep tag and encoded by the pSMT3 vector together with *pe35*, *esxB*, and *esxA*. Immunoblot analysis of the pellet fractions using the anti-Strep antibody showed that the two more severe C-terminal truncations of PPE68 were detected in lower amounts, and only the PPE68Δ10 variant was produced at similar levels as the full-length protein (Fig. 2B, lanes 4 to 6). The anti-PPE68 antibody could only recognize the PPE68Δ10 variant, indicating that the antibody primarily recognized the C-terminal domain of this protein. Both PPE68Δ122 and PPE68Δ10 were able to restore EsxA secretion by the *ppe68* operon mutant (Fig. 2B, lanes 11 and 12), whereas with the PPE68ΔC variant EsxA secretion could hardly be observed, most likely because this variant was not stably produced (Fig. 2B, lane 10). In contrast, while the intracellular protein levels of EspE were similar between all strains, none of the PPE68 variants showed detectable secretion of EspE (Fig. 2B, lanes 4 to 6 and 10 to 12). These results suggest that the C-terminal domain of PPE68 is not required for EsxA secretion, but it is necessary for the secretion of EspE.

### PPE68 is secreted independently from EsxA and EsxB and processed on the cell surface.

**FIG 3:**
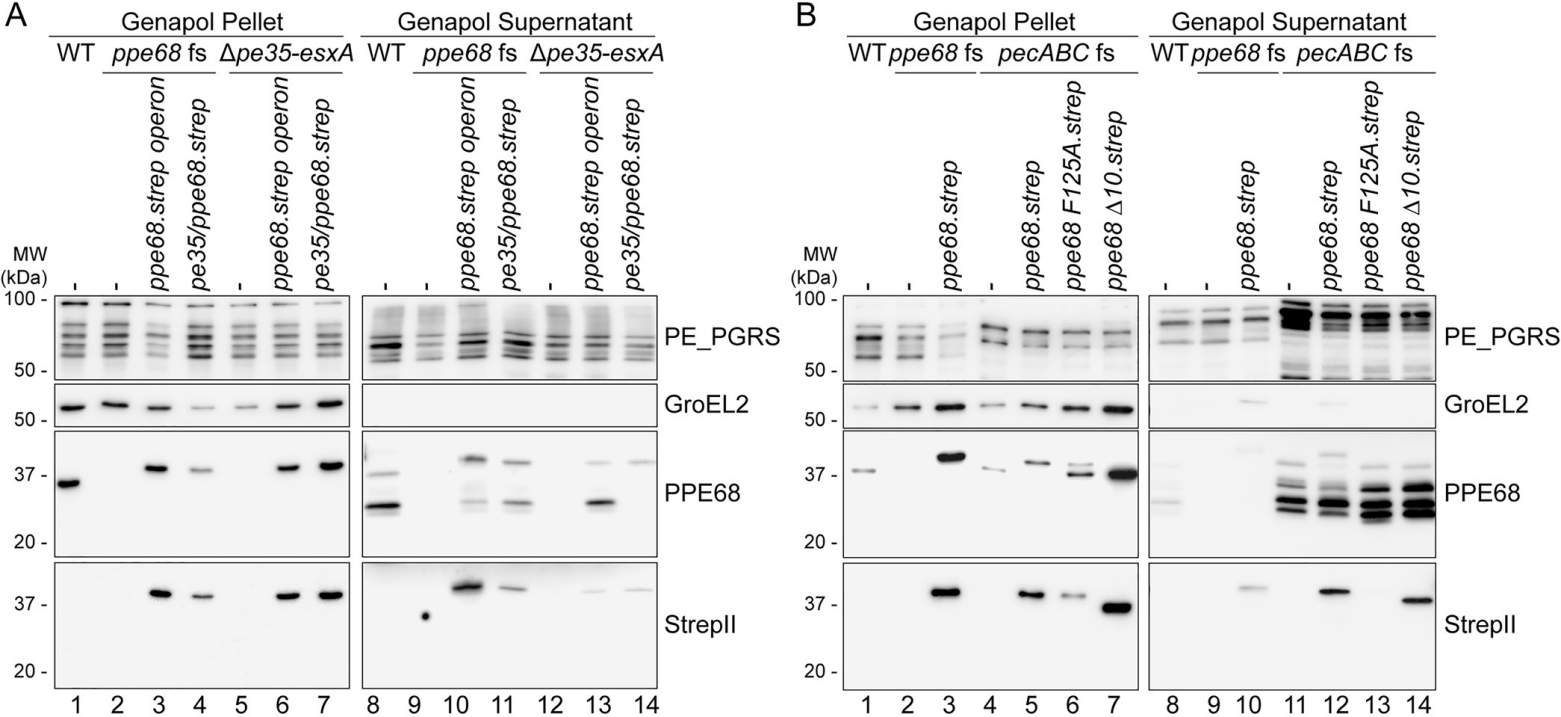
PPE68 is secreted independently of *esxB* and *esxA* and processed on the cell surface of M. marinum. (A) SDS-PAGE and immunostaining on Genapol pellet and concentrated Genapol supernatant fractions of M. marinum WT, the *ppe68* fs mutant, the *ppe68* operon mutant, or both mutants complemented with *pe35/ppe68* or the entire *ppe68* operon. (B) Additional SDS-PAGE and immunostaining of Genapol pellet and concentrated Genapol supernatant fractions of the *pecABC* fs mutant overexpressing the WT *ppe68* operon (*ppe68.strep*), the operon producing either the PPE68F125A with an amino acid substitution in the helical tip, PPE68Δ10 with a C-terminal truncation of the last 10 amino acids. Operons were expressed from a multicopy pSMT3 vector controlled by the *hsp60* promoter and with a Strep tag on the C terminus of PPE68 and the variants. Proteins were visualized with anti-StrepII (PPE68) and anti-PPE68 antibodies (ESX-1 substrates). All *ppe68* fs and *pecABC* fs mutants contained an additional pMV TdTomato plasmid. As a loading and lysis control, blots were incubated with antibodies directed against the cytosolic GroEL2 protein and the surface-localized PE_PGRS proteins. In all blots, equivalent OD units were loaded: 0.2 OD for Genapol pellet and 2 OD Genapol supernatant fractions. Data shown in each panel are representative of three independent experiments.

Although PPE68 contains all characteristics of an ESX substrate, we were not able to detect this protein in culture supernatants (Fig. 1 and 2). However, when we obtained a concentrated cell surface protein extract from larger cultures that were additionally grown without Tween 80, which has been shown to retain surface proteins (52), low levels of PPE68 were detected in the surface extract of WT M. marinum (Fig. 3A, lane 8). Here, PPE68 seemed to be processed, as multiple bands were recognized by the anti-PPE68 antibody. Only full-length PPE68 was observed by the anti-StrepII antibody, suggesting that the other bands detected by the anti-PPE68 antibody represented C-terminal truncates. C-terminal processing of the Strep tag was also observed for the secreted fraction of the PPE68_1/PPE68 hybrid (Fig. 2A). As we were now able to detect surface-localized PPE68, we analyzed whether the export of PPE68 was dependent on the coexpression of *esxB* and *esx*A. Importantly, a pSMT3 vector with only the *pe35* and *ppe68.strep* genes fully restored PPE68 secretion to the cell surface by the two *ppe68* mutants (Fig. 3A, lanes 11 and 14). As the *ppe68* operon mutant did not contain a genomic copy of *esxB* or *esxA*, these results suggested that PPE68 can be secreted independently of EsxA (Fig. 3A, lane 14). Intriguingly, only full-length PPE68.strep was detected on the cell surface of the complemented *ppe68* operon mutant, suggesting that surface processing of PPE68 might be dependent on the cosecretion of EsxA.

The various processed forms of PPE68 on the surface indicated that this protein is degraded upon secretion. Previously, the aspartic protease PecA was shown to cleave PE_PGRS proteins on the cell surface of M. marinum (18). We therefore hypothesized that this protein, together with its paralogs PecB and PecC, could be involved in cell surface processing of PPE68. Using a *pecA-pecB-pecC* triple-fs mutant, created using CRISPR1-Cas9 gene editing (unpublished data), we could confirm that, while PPE68 levels were similar in the Genapol pellet fractions (Fig. 3B, lanes 1 and 4), the amount of PPE68 protein that was detected in the upscaled cell surface extract was significantly higher in the *pecABC* fs mutant compared to that of the WT (Fig. 3B, lanes 8 and 11). This showed that the amount of exported PPE68 was in fact higher than previously assumed. As similar processing of surface-localized PPE68 was still observed in the *pecABC* fs mutant, another protease should be involved in this process. A prime candidate would be the serine protease MycP_1_, which is one of the conserved ESX-1 components. However, a point mutation in the active site of MycP_1_ (53) did not affect the surface processing of PPE68 (Fig. S4).

10.1128/mbio.02819-22.4 FIG S4 A point mutation in the active site of serine protease MycP_1_ does not affect the processing of PPE68 on the cell surface. SDS-PAGE and immunostaining of concentrated Genapol supernatant fractions of M. marinum WT and a *mycP_1_* deletion mutant complemented with a *mycP_1_* active site (S354A) variant, expressed from a pMV vector by the *hsp60* promotor. Proteins were visualized with an anti-PPE68 antibody. As a loading and lysis control, blots were incubated with antibodies directed against the cytosolic GroEL2 protein and the surface-localized PE_PGRS proteins. In all blots, equivalent amounts, i.e. 2 OD units, of Genapol supernatant fractions were loaded. Data shown are representative of two independent experiments. Download FIG S4, TIF file, 0.6 MB. Copyright © 2022 Damen et al. 2022 Damen et al. https://creativecommons.org/licenses/by/4.0/ This content is distributed under the terms of the Creative Commons Attribution 4.0 International license.

Next, we exploited the increased amount of cell surface-localized PPE68 in the *pecABC* fs mutant to further investigate the requirement for PPE68 secretion. When we disrupted the interaction with EspG_1_ by the F125A mutation, PPE68.strep was not detected in the surface fraction (Fig. 3B, lane 13), confirming the importance of the EspG_1_ interaction for secretion. Interestingly, deletion of the C-terminal 10 amino acids of PPE68, which did not majorly affect EsxA secretion but abrogated secretion of EspE, did not interfere with secretion of PPE68 itself (Fig. 3B, lane 14). To summarize, PPE68 is exported to the cell surface of M. marinum, where it is subsequently degraded. Secretion of PPE68 depends on its interaction with EspG_1_ but is independent of coexpression of *esxB* and *esxA* and its final 10 amino acids.

### Intracellular PPE68 forms a complex with EspG_1_ and a PE protein.

**FIG 4:**
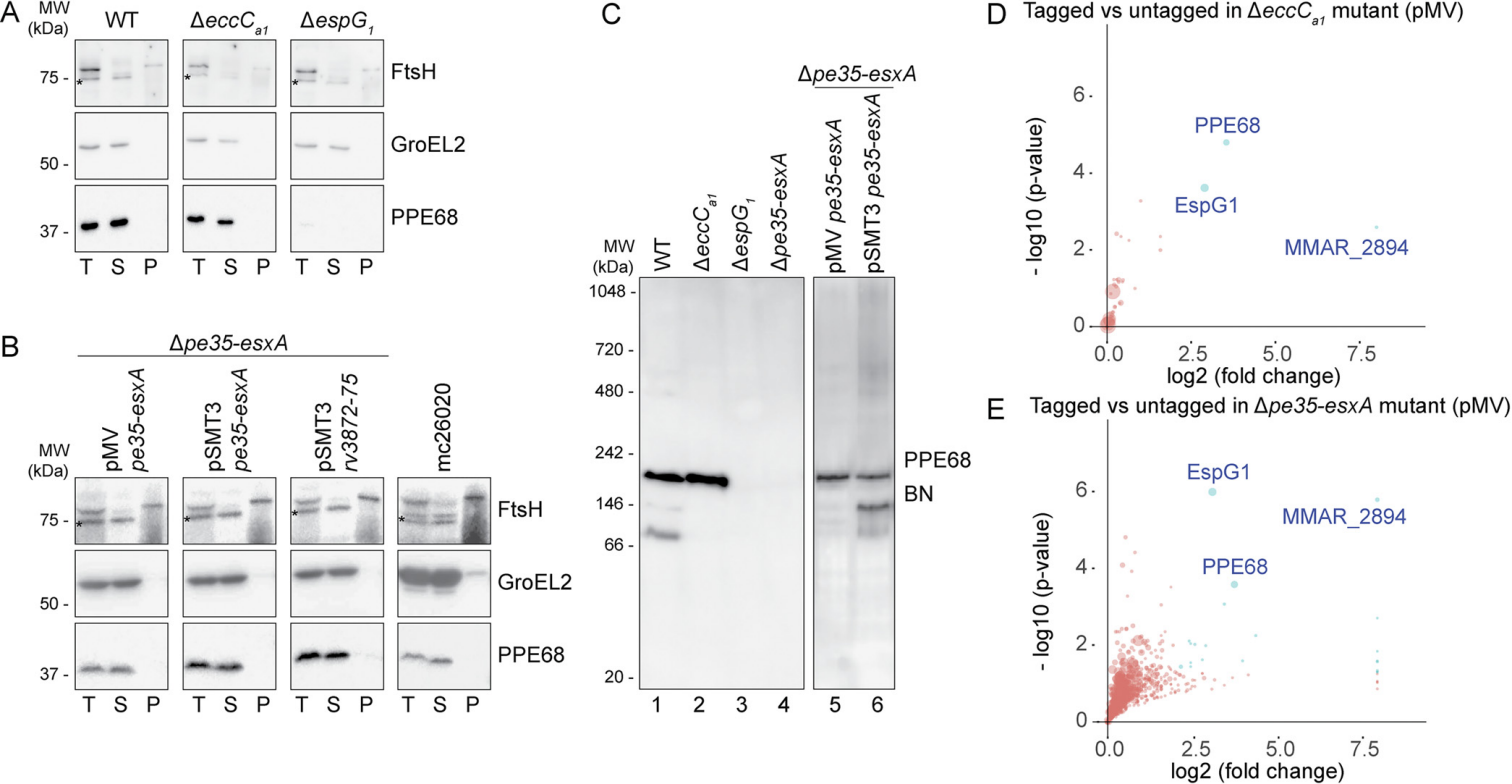
PPE68 of M. marinum and M. tuberculosis is soluble and forms complexes of different sizes. (A and B) SDS-PAGE and immunostaining of subcellular fractionation of M. marinum WT, an *eccC_a1_* mutant, and an *espG_1_* mutant (A) or the *ppe68* operon mutant complemented with the *ppe68* operon of M. marinum expressed from a pMV or pSMT3 vector, the *ppe68* operon of M. tuberculosis H37Rv expressed from a pSMT3 vector, or auxotrophic M. tuberculosis *strain* mc^2^6020 (B). Proteins were visualized with anti-PPE68, while antibodies against GroEL2 (cytosolic protein) and FtsH (membrane protein) were used to assess the purity of the fractions. Notably, the FtsH antiserum cross-reacted with a cytosolic protein (indicated by an asterisk). In all blots, equivalent amounts corresponding to 0.2 OD units were loaded. T, total lysate; S, supernatant or soluble proteins; P, pellet or cell envelope proteins. (C) Immunostaining of soluble fractions of the same strains as in panels A and B after blue native PAGE (BN-PAGE) gel electrophoresis. In all blots, an equivalent amount of 19 μg of total protein was loaded. Data shown in panels A, B, and C are representative of two independent experiments. (D and E) PPE68 interacts with its PE partner, MMAR_2894, and the EspG_1_ chaperone in the cytosol. Quantitative proteomics analysis comparing the PPE68 affinity purification from the soluble fraction of the *eccC_a1_* mutant (D) and the *ppe68* operon mutant (E), complemented with the *ppe68* operon, produced PPE68 with or without a Strep tag on its C terminus from a pMV vector. Proteins that showed a log_2_ fold change of >2 and a −log_10_ *P* value of >1.3 between tagged and untagged PPE68 are indicated as blue dots. Sizes of the dots are correlated to the number of spectral counts. Data include two technical replicates per sample.

While small amounts of PPE68 could be extracted from the cell surface, a large portion of PPE68 remained cell associated upon detergent treatment (Fig. 3). In M. tuberculosis, PPE68 was shown to localize to the cell envelope fraction by subcellular fractionations (9, 48). However, when we fractionated M. marinum cells by high-pressure lysis followed by ultracentrifugation, we found the entire population of intracellular PPE68 to be present in the soluble fraction (Fig. 4A). In an *eccC_a1_* deletion strain, production and solubility of PPE68 were similar to those in the WT, while deleting the *espG_1_* chaperone gene drastically reduced the amount of PPE68. Exogenous expression of the entire *ppe68* operon in the *ppe68* operon mutant did not alter the solubility of PPE68 (Fig. 4B). To test whether the observed difference in solubility between PPE68 homologs was species specific or dependent on experimental procedures, we fractionated M. marinum expressing the *ppe68* operon of M. tuberculosis and the auxotrophic M. tuberculosis strain mc^2^6020 using the same procedure. In both cases, PPE68 of M. tuberculosis was again exclusively present in the soluble fraction (Fig. 4B); from this we conclude that the observed differences for PPE68 localization are a result of different protocols for cell lysis and subcellular fractionation.

The current paradigm is that PPE proteins interact with a specific PE partner and a system-specific EspG chaperone in the cytosol prior to secretion (12, 13, 54). Analysis of PPE68 complex formation by blue native PAGE (BN-PAGE) revealed three major intracellular complexes ranging from ~70 to 180 kDa (Fig. 4C, lane 1). Both the *ppe68* operon mutant and the Δ*espG_1_* mutant did not show any PPE68 complex formation (Fig. 4C, lanes 3 and 4). In the soluble fraction of the Δ*eccC_a1_* mutant, in which export of PPE68 is blocked, the largest (~180-kDa) and predominant complex was observed in similar amounts as in the WT fraction (Fig. 4C, lane 2), suggesting that a large portion of PPE68 in WT bacteria is retained in the cytosol. The additional two complexes were absent in the Δ*eccC_a1_* mutant, indicating that these two assemblies are only formed in the presence of a functional ESX-1 membrane complex. Complementation of the *ppe68* operon mutant with the *ppe68* operon of M. marinum, either using the pMV or pSMT3 plasmid, restored formation of the ~180-kDa PPE68 complex, while the two smaller complexes were only clearly observed when complemented with the pSMT3 vector (Fig. 4C, lanes 5 and 6). This difference could be due to higher expression levels of the operon genes of the latter vector, compared to that of the integrative pMV vector.

To analyze the composition of the observed complexes, a Strep affinity purification was performed on the soluble fractions of the Δ*eccC_a1_* and *ppe68* operon mutants, producing Strep-tagged PPE68 from either the pMV or pSMT3 vector containing the full *ppe68* operon. Both mutants containing the *ppe68* operon without a Strep tag sequence were included as negative controls. BN-PAGE analysis of the purified samples showed a similar pattern of PPE68 complexes as seen for endogenous PPE68 (Fig. S5C). Liquid chromatography-tandem mass spectrometry (LC-MS/MS) analysis showed that the PPE68.strep samples, purified from the Δ*eccC_a1_* mutant, contained, as expected, PPE68 and the chaperone EspG_1_. The third protein that was present in high amounts was an M. marinum-specific PE protein, named MMAR_2894 (Fig. 4D and Table S1). Interestingly, this protein was already identified as an ESX-1 substrate (46). Notably, PE35 of M. marinum, unlike its ortholog in M. tuberculosis, lacks a general YxxxD/E secretion motif (Fig. S6) (15), which might explain why PPE68 binds an alternative partner in M. marinum. As PE35_1 was also not detected, MMAR_2894 seems to be the only PE partner of PPE68 in M. marinum. EspG_1_ and MMAR_2894 were also the predominant copurified proteins of PPE68 purified from the complemented *ppe68* operon mutant, confirming that intracellular PPE68 localized to the cytosol also in the presence of a functional ESX-1 system (Fig. 4E and Table S1). PPE68.strep produced from the pSMT3 vector in both the *ppe68* operon and Δ*eccC_a1_* mutant also showed the specific copurification of EspG_1_ and MMAR_2894 (Fig. S5D and E).

10.1128/mbio.02819-22.5 FIG S5 Purification of PPE68 from the *ppe68* operon mutant and the *eccC_a1_* mutant containing a pMV or pSMT3 vector with the *ppe68* operon of M. marinum. Genes were expressed from the *hsp60* promotor with or without a Strep tag on the C terminus of PPE68. (A) Immunostaining of subcellular fractionations of the different strains. Proteins were visualized with anti-PPE68 and anti-StrepII antibodies, while anti-FtsH antibodies controlled for the loading and lysis. Notably, the FtsH antiserum cross-reacted with a cytosolic protein. In all blots, equivalent amounts corresponding to 0.2 OD units were loaded. T, total lysate; S, supernatant or soluble proteins; P, pellet or cell envelope proteins. (B) Separation of the PPE68 purification samples by SDS-PAGE and subsequent staining with Coomassie brilliant blue. (C) Separation of complexes in the PPE68 purification samples by BN-PAGE and subsequent staining with Coomassie brilliant blue. As a negative control, the purification sample of the *ppe68* operon mutant containing a pSMT3 vector with the *ppe68* operon, without C-terminal Strep tag on PPE68, was included. Data shown in panls A, B, and C are representative of two independent experiments. (D) Quantitative proteomics analysis comparing the PPE68 affinity purification from the soluble fraction of the Δ*eccC_a1_* mutant and the *ppe68* operon mutant (Δ*pe35-esxA*) producing PPE68 with or without a Strep tag on the C terminus, from a pSMT3 vector. Data included two technical replicates per sample. Proteins that showed a log2 fold change of >2 and a −log10 *P* value of >1.3 between tagged and untagged PPE68 are indicated as blue dots. Size of the dots are correlated to the number of spectral counts. Download FIG S5, TIF file, 1.4 MB. Copyright © 2022 Damen et al. 2022 Damen et al. https://creativecommons.org/licenses/by/4.0/ This content is distributed under the terms of the Creative Commons Attribution 4.0 International license.

10.1128/mbio.02819-22.6 FIG S6 Protein sequence alignment of PPE35 homologs of M. marinum and M. tuberculosis, including the secondary structure prediction of M. tuberculosis PE35. The secondary structure prediction was produced by Alphafold2. The blue square indicates the conserved YxxxD/E secretion motif. Note that PE35 of M. marinum lacks this conserved motif. Rv3872, PE35 of M. tuberculosis; PE35, MMAR_5447 of M. marinum; PE35_1, MMAR_0185 of M. marinum. Download FIG S6, TIF file, 1.3 MB. Copyright © 2022 Damen et al. 2022 Damen et al. https://creativecommons.org/licenses/by/4.0/ This content is distributed under the terms of the Creative Commons Attribution 4.0 International license.

10.1128/mbio.02819-22.8 TABLE S1 Significant hits of PPE68 interactors as determined by protein purification and mass spectrometry analysis (only proteins with an average normalized spectral count of >5 in the tagged samples, a log2 fold change of >2, and a −log10 *P* value of >1.3 between tagged and untagged samples are shown). Download Table S1, DOCX file, 0.01 MB. Copyright © 2022 Damen et al. 2022 Damen et al. https://creativecommons.org/licenses/by/4.0/ This content is distributed under the terms of the Creative Commons Attribution 4.0 International license.

**FIG 5:**
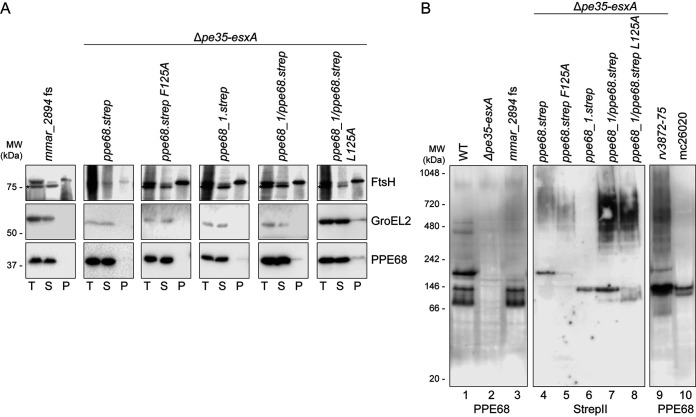
Verification of complex formation of PPE68 with MMAR_2894 and EspG_1_. (A) SDS-PAGE and immunostaining of subcellular fractions of the *mmar_2894* fs mutant and the *ppe68* operon mutant complemented with the WT *ppe68* operon, the *ppe68_1* operon, a hybrid operon containing *pe35_1*, the N-terminal domain of *ppe68_1*, the C-terminal domain of *ppe68*, and *esxB* and *esxA* or the *ppe68* or hybrid operon expressing a PPE68 variant with a single amino acid substitution at position 125. Genes were expressed from a multicopy pSMT3 vector under the *hsp60* promoter with a Strep tag on the C terminus of PPE68 or its variants. Proteins were visualized with anti-PPE68 antibodies, while antibodies against GroEL2 (cytosolic protein) and FtsH (membrane protein) were used to assess the purity of the fractions. Notably, the FtsH antiserum cross-reacted with a cytosolic protein (indicated by an asterisk). In all blots, equivalent amounts corresponding to 0.2 OD units were loaded. T, total lysate; S, supernatant or soluble proteins; P, pellet or cell envelope proteins. (B) BN-PAGE and immunostaining of soluble fractions of the same strains as in panel A, and also the *ppe68* operon mutant expressing the *ppe68* operon of M. tuberculosis from a pSMT3 vector and the auxotrophic M. tuberculosis strain mc^2^6020. Proteins were visualized with anti-PPE68 and anti-StrepII antibodies. In all blots, an equivalent amount of 19 μg of total protein was loaded. Data shown in each panel are representative of two independent experiments.

To confirm the presence of MMAR_2894 and EspG_1_ in the PPE68 complexes, we created an *mmar_2894* fs mutant and analyzed the PPE68F125A variant, respectively. The *mmar_2894* fs mutant, which contained a 13-bp deletion after 192 bp that resulted in an early stop codon after 68 amino acids (Fig. S1B), showed an ESX-1 secretion defect and loss of hemolytic capabilities similar to the *ppe68* fs mutant (Fig. S7A to C). While PPE68 was detectable and soluble in the *mmar_2894* fs mutant, BN-PAGE analysis showed that the major ~180-kDa complex was absent in this mutant. The smaller two complexes were still present, suggesting that only the predominant complex contained MMAR_2894 (Fig. 5B, lane 3). With the PPE68F125A variant, no complexes could be observed by BN-PAGE analysis (Fig. 5B, lane 5), while it was detectable and in the soluble fraction by SDS-PAGE analysis, suggesting that all three complexes contained EspG_1_.

10.1128/mbio.02819-22.7 FIG S7 Secretion and hemolytic analysis of the *mmar_2894* fs mutant. (A) SDS-PAGE and immunostaining of the cell pellet, culture supernatant, Genapol pellet, and Genapol supernatant fractions of M. marinum, WT, an ESX-1 mutant (*eccC_b1_* mutant; M^VU^), and the *mmar_2894* fs mutant. Proteins were visualized with anti-EsxA, anti-EspE, and anti-PPE68 antibodies (ESX-1 substrates). A processed band of 25 kDa was detected by the anti-EspE antibody in the Genapol supernatant fraction of the WT, which has been reported before (Phan et al., 2018 [46]). As a loading and lysis control, blots were incubated with antibodies directed against the cytosolic GroEL2 protein. In all blots, equivalent OD units were loaded: 0.2 OD for pellet and Genapol pellet and 0.4 OD for supernatant and Genapol supernatant fractions. (B) SDS-PAGE and immunostaining of the Genapol pellet and upscaled Genapol supernatant fractions of M. marinum WT, the *ppe68* fs mutant, and the *mmar_2894* fs mutant. Proteins were visualized with an anti-PPE68 antibody (ESX-1 substrate). As a loading and lysis control, blots were incubated with antibodies directed against the cytosolic GroEL2 protein and the surface-localized PE_PGRS proteins. In all blots, equivalent OD units were loaded: 0.2 OD for Genapol pellet and 2 OD Genapol supernatant fractions. Data shown in panels A and B are representative of two independent experiments. (C) Hemoglobin release after incubation of the same strains shown in panels A and B with sheep red blood cells, quantified by determining the OD_450_ of the medium after incubation. Data shown are from two independent experiments that included 3 technical replicates each. Download FIG S7, TIF file, 1.4 MB. Copyright © 2022 Damen et al. 2022 Damen et al. https://creativecommons.org/licenses/by/4.0/ This content is distributed under the terms of the Creative Commons Attribution 4.0 International license.

Finally, we also analyzed complex formation of PPE68 from M. tuberculosis using both heterologous expression of the *ppe68* operon in M. marinum and the auxotrophic M. tuberculosis strain mc^2^6020. The observed PPE68 complexes in both strains had the same size as PPE68_1.strep and the PPE68_1/PPE68.strep hybrid (Fig. 5B, lane 9 and 10). The smaller sizes of these complexes compared to the ~180-kDa PPE68_mmar_ complex could be due to the sizes of their most likely PE partners, i.e., PE35_1 and PE35_mtub_, respectively, which are smaller (9 kDa) than MMAR_2894 (22 kDa). Structural analysis suggests that PE-PPE-EspG complexes are formed by a single copy of each protein (12, 13), resulting in a molecular weight for MMAR_2894-PPE68-EspG_1_ of M. marinum and both PE35_1-PPE68_1-EspG_1_ of M. marinum and PE35-PPE68-EspG_1_ of M. tuberculosis of 89 kDa and 74 kDa, respectively. The fact that the observed complexes had twice the molecular weight, i.e., ~180 kDa and ~150 kDa, respectively, indicated that the PE-PPE-EspG complexes form a dimer of trimers in the cytosol of mycobacteria. In summary, our fractionation and pulldown results suggest that cell-associated PPE68 of M. marinum and M. tuberculosis is mainly located in the cytosol, where it is in a complex together with EspG_1_ and its PE partner.

### Role of PPE68 in ESX-1 secretion: a model.

**FIG 6:**
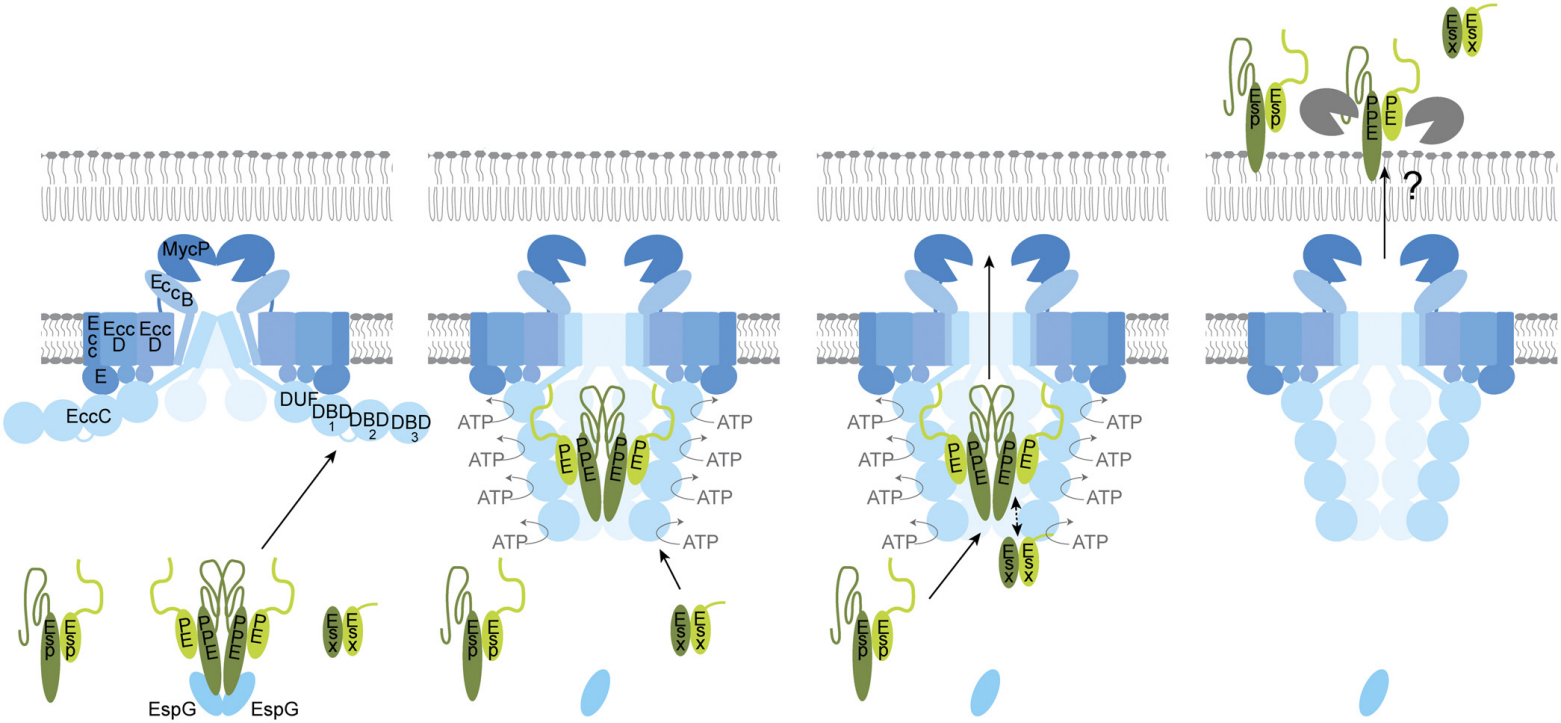
Working model for the role of PPE68 in ESX-1-mediated secretion. We propose that different ESX-1 substrates are secreted in a hierarchical fashion by a stepwise interaction with the secretion machinery. Here, the PE/PPE proteins activate the channel by interacting with the linker that connects NBD1 and NBD2 of the central EccC component. This interaction induces conformational changes in the ATPase and its full activation, after which the Esx substrates can bind NBD3, resulting in the cosecretion of the PE/PPE and Esx substrates across the cell envelope. The secretion of both the PE/PPE and Esx heterodimers is necessary for secretion of the final substrate family, the Esp proteins.

Based on our results, we propose a working model for PPE68 in the secretion of EsxA and EspE via the ESX-1 secretion system in M. marinum (Fig. 6). In the cytosol, PPE68 forms a stable complex together with its PE partner, i.e., MMAR_2894 in M. marinum, and chaperone EspG_1_. Upon targeting to the ESX-1 membrane complex, the PE/PPE pair binds to the central ATPase EccC_ab1_, possibly to the linker 2 domain between nucleotide-binding domain 1 (NBD1) and NBD2, a previously proposed recognition site for PE/PPE substrates (55). Based on our observation that PPE68 does not require EsxA/EsxB for its secretion, this binding event is enough to trigger activation of the translocation channel. As a second step, EsxA/EsxB binds, based on *in vitro* binding studies and structural analysis, to NBD3 of EccC_ab1_ (26, 56–58). The Esx pair probably also interacts with the PE/PPE pair at the membrane complex, as PE35_1/PPE68_1 has been shown to determine the system specificity of EsxB_1/EsxA_1 (43). As the third step, EspE is targeted with its predicted partner EspF and chaperone EspH (46) to the same ESX-1 complex. Secretion of the PE/PPE and Esx pairs probably does not depend on EspE, as mutations in EspH only affect secretion of EspE and EspF (46). Subsequently, the three substrate pairs are transported together through the channel at the cost of ATP hydrolysis by the EccC ATPases. EspE interacts with the C terminus of PPE68 during the translocation process, as these residues are important for the secretion of this Esp substrate specifically. The mechanism of translocation across the mycobacterial outer membrane remains unknown. While it is tempting to speculate that PPE68 plays a role in the process, the protein does not form a stable channel in this specific membrane. Instead, the main portion of PPE68 exists as a cytosolic pool, where it serves as a reservoir to facilitate secretion of the other ESX-1 substrates. Once PPE68 is exported to the cell surface, it is processed and/or degraded by PecABC and an unspecified protease.

## DISCUSSION

The ESX-1 substrate EsxA is an abundantly secreted and highly immunogenic protein of M. tuberculosis and M. marinum (59–61) and has therefore been a major focus to get mechanistic insights into the essential role of ESX-1 in phagosomal membrane rupture by these pathogens (3, 4, 6, 24–26). However, the investigation of EsxA, or other ESX-1 substrates, remains difficult due to the codependent secretion of these substrates (34, 36–39). Here, we report that ESX-1 substrate PPE68, which is encoded within the same operon as EsxA, plays a central role in the secretion of EsxA and EspE in M. marinum, and we provide mechanistic insights into the observed codependent secretion of these substrates.

First, we showed that PPE68 is exported in an ESX-1-dependent manner to the cell surface of M. marinum. While previous mass spectrometry analysis could also detect this protein in culture supernatants of M. marinum (33, 46, 62), we could not detect PPE68 in similar fractions by immunoblot analysis. Intriguingly, this protein is degraded upon secretion to the cell surface, as deletion of three Pec proteases (PecABC) increased the amount of PPE68 on the cell surface. This provided an explanation for the small amount of exported PPE68 we detected. The observation that the *pecABC* deletions did not affect the processing pattern of PPE68 protein suggested that an additional protease is involved in its processing and/or degradation.

A frameshift mutation in *ppe68* abolished secretion of EsxA and EspE, and of hemolytic activity by M. marinum, which could be fully complemented by introduction of the *pe35*-*ppe68*-*esxB*-*esxA* operon. The role of PPE68 in the secretion process depended on the export of the PPE protein, as a point mutation in its EspG_1_-binding domain that blocked PPE export abolished secretion of the other ESX-1 substrates. Importantly, PPE68 export was independent of *esxA*/*esxB* expression, showing there is a hierarchy in codependent secretion. While the Esx pair has been considered to be important for secretion of all other ESX substrates, our results confirmed recently published data showing that MMAR_2894, the PE partner of PPE68 in M. marinum, is fully independent of *esxB*/*esxA* for its export (62).

Notably, we showed that PPE68 of M. tuberculosis can take over the central role of endogenous PPE68 of M. marinum in ESX-1-mediated secretion and hemolysis. Our observations and those made by Cronin et al. in M. marinum contrast results from previous studies in M. tuberculosis complex members that showed that interruption of the *ppe68* gene did not affect secretion of EsxA in RD1-complemented Mycobacterium microti, M. bovis BCG, or M. tuberculosis H37Rv (48, 63). In the same studies, the *ppe68* deletion in RD1-complemented *M. microti* and M. bovis BCG also did not decrease virulence in severe combined immunodeficient mice compared to the WT-complemented strains. However, in other studies, *ppe68* transposon mutants did affect growth of M. tuberculosis H37Rv in a C57BL/6J mouse infection model (64) and also led to reduced cytotoxicity in THP-1 macrophages (65). Interestingly, host cell cytolysis, hemolytic activity, and *in vivo* virulence phenotypes of *esx-1* mutants do not necessarily correlate, as reported in multiple studies (46, 62, 66). While the reason for the observed differences in requirements for EsxA secretion between M. marinum and M. tuberculosis remains unclear, these differences might be related to the different PE partner for PPE68 and/or the duplication of the *pe35-esxA* region in M. marinum. Alternatively, they might be caused by a variation in *in vitro* growth conditions, as previously observed for the importance of the conserved cytosolic component EccA_1_ in secretion (46). While analyzing a similar *ppe68* fs mutant of M. tuberculosis would address the seemingly species-specific differences, our observations using the auxotrophic M. tuberculosis strain mc^2^6020 and the M. marinum *ppe68* fs mutant complemented with the *ppe68* operon of M. tuberculosis showed that at least the subcellular location of PPE68 is conserved and that the M. tuberculosis protein can take over the essential role of its ortholog in ESX-1-mediated secretion in M. marinum.

A codependent relationship between substrates of the same ESX system could be explained by a more integral role of specific substrates in the translocation process itself. Recently, Cronin et al. proposed a model in which MMAR_2894/PPE68 forms the periplasmic part of the ESX-1 channel and EsxA/EsxB mediates translocation of ESX-1 substrates across the mycobacterial outer membrane (62). This model is supported by a previous observation that PPE68 of M. tuberculosis localizes to cell envelope fractions (9, 48). However, we observed that cell-associated PPE68 was soluble, both in M. marinum and the auxotrophic M. tuberculosis strain mc^2^6020. These contrasting observations were probably caused by experimental differences. In addition, our observation that PPE68 still localized to the cell surface in the absence of *esxB*/*esxA* argues against a role of the Esx pair in outer membrane translocation. Although a function for PPE68 in outer membrane translocation is still possible, the fact that we found this protein predominantly in the cytoplasmic compartment points toward a role in substrate recognition and/or activation by the ESX-1 inner membrane complex. Our previous observation that PPE68_1 determines through which ESX system EsxA_1 is exported by M. marinum is in agreement with such a function (43). In addition, a domain in the EccC ATPase component, i.e., linker 2 between NBD1 and NBD2, has previously been shown to be involved in species-specific secretion of PE_PGRS proteins via the ESX-5 system (55). We therefore propose that binding of the PE/PPE pair to linker 2 on EccC_ab1_ could trigger the activation of the ATPases and the opening of the ESX inner membrane complex.

Interestingly, we observed that the final 112 amino acids of PPE68 are dispensable for EsxA secretion. This suggested that the conserved N-terminal PPE domain of this protein, probably together with the PE domain of its partner, is involved in the secretion of other ESX-1 substrates. This involvement could be indirect, by activation of the ESX membrane channel, but it could also be mediated by a direct interaction of the two substrate pairs. In contrast, the C terminus of PPE68 seemed to be required for the secretion of EspE, i.e., deletion of only the C-terminal 10 amino acids already blocked EspE secretion. This charged C-terminal tail was conserved in the PPE68 homolog of M. tuberculosis and the PPE68_1 paralog in M. marinum, possibly explaining why these homologs are able to complement EspE secretion in the M. marinum *ppe68* mutants. We propose that EspE interacts with the C-terminal domain of PPE68 during the secretion process.

The major portion of cytosolic PPE68 is in an ~180-kDa complex with the EspG_1_ chaperone and an unexpected PE protein, namely, MMAR_2894. MMAR_2894 has previously been identified as an ESX-1 substrate with a role in hemolysis (46, 66). Mutating *mmar_2894* leads to the absence of the major cytosolic PPE68 complex and a secretion and hemolytic defect identical to that in the *ppe68* fs mutant, which confirms that MMAR_2894 is the PE partner of PPE68 in M. marinum. This also means that PPE68 does not pair with the adjacently encoded PE35, which explains why PE35 lacks a T7SS signal (YxxxD/E). Apparently, during evolution PE35 was replaced by MMAR_2894 and lost its function, or vice versa. Analysis of multiple available M. marinum genomes (67) revealed a widespread lack of the T7SS signal in PE35, which extended to its close relatives Mycobacterium liflandii and Mycobacterium ulcerans, suggesting that this variation arose in a common ancestor. It seems that new associations between PE and PPE proteins can be formed, which also cautions the interpretation of adjacent PE and PPE genes as necessary secretion partners. Based on available structural data on PE:PPE:EspG complexes and observed molecular weights of the major cytosolic complex, we postulate that the MMAR_2894:PPE68:EspG_1_ complex forms a dimer of trimers. Dimers of EspG_3_ from M. tuberculosis and Mycobacterium smegmatis have been crystallized, suggesting that dimerization could occur via the chaperone (12, 68).

Overall, our study describes a central role for PPE68 in ESX-1-mediated secretion and function. We hypothesize that PPE68 is in complex together with its PE partner and EspG chaperone in the cytosol as a reservoir to aid in the secretion of other substrates across the inner and possibly also the outer membrane. Further studies should focus on the exact mechanism of codependence of substrates for secretion, potential interactions between secreted codependent heterodimers, and the role of the EccC ATPase in this process. The recent publications of the high-resolution structure of various ESX systems (69–72) will stimulate the field toward a mechanistic understanding of T7SSs.

## MATERIALS AND METHODS

### Bacterial strains and culture conditions.

All strains included in this study are listed in Table S2 in the supplemental material. Mycobacterial strains were routinely grown on Middlebrook 7H10 agar with 10% oleic acid-albumin-dextrose-catalase (OADC; Difco) or in Middlebrook 7H9 liquid medium with 10% ADC (Difco) and 0.05% Tween 80. Additional supplement was necessary for culturing of M. tuberculosis mc^2^6020 (Δ*lysA* Δ*panCD*), for which 100 ng/mL l-lysine (Sigma) and 25 ng/mL d-pantothenic acid (Sigma) were included. Appropriate antibiotics were added when necessary, i.e., 50 μg/mL kanamycin (Sigma), 50 μg/mL hygromycin (Roche), or 30 μg/mL streptomycin (Sigma). M. marinum and M. tuberculosis cultures and plates were incubated at 30°C and 37°C, respectively. Escherichia coli DH5α was used for cloning and was grown on LB agar plates at 37°C. Antibiotics were added at the same concentrations as described above.

10.1128/mbio.02819-22.9 TABLE S2 Strains and plasmids used in this study. Download Table S2, DOCX file, 0.03 MB. Copyright © 2022 Damen et al. 2022 Damen et al. https://creativecommons.org/licenses/by/4.0/ This content is distributed under the terms of the Creative Commons Attribution 4.0 International license.

### Plasmid construction.

Genes of interest were amplified from M. marinum M or M. tuberculosis H37Rv genomic DNA by PCR using primers that were synthesized by Sigma (Table S3). Hybrid combinations of genes or introduction of affinity tags was achieved with the use of nested primers. The Phusion polymerase (New England Biolabs [NEB]) was used throughout all cloning procedures. Generated constructs were digested with XmnI and HindIII or NheI and BamHI for ligation into pMV361 and pSMT3 vectors (73, 74), respectively. Restriction enzymes and ligation enzymes were provided by NEB. All plasmids were verified by Sanger sequencing (Macrogen) and are listed in Table S2.

10.1128/mbio.02819-22.10 TABLE S3 Primers used in this study. Download Table S3, DOCX file, 0.02 MB. Copyright © 2022 Damen et al. 2022 Damen et al. https://creativecommons.org/licenses/by/4.0/ This content is distributed under the terms of the Creative Commons Attribution 4.0 International license.

### Generation of knockout strains and frameshift mutants.

Generation of the *mmar_5447-50* and *mmar_0185-88* knockout strains was performed in M. marinum M by allelic exchange using a specialized transducing mycobacteriophage as previously described (75). PCR was used to amplify the flanking regions of the genomic regions. Deletions were confirmed by PCR, after which the selection markers were removed with a temperature-sensitive phage encoding the γδ-resolvase (TnpR; a kind gift from Apoorva Bhatt, University of Birmingham, United Kingdom). The fs mutants were obtained by genome editing using Streptococcus thermophilus CRISPR1-Cas9 (47). Single-guide RNAs (sgRNAs) were produced by the annealing of oligonucleotides containing BsmBI overhangs (76). sgRNAs were ligated into BsmBI-digested pCRISPRx-Sth1Cas9-L5. The plasmids were sequenced and subsequently electroporated into competent M. marinum M cells. Bacteria were plated on 7H10 plates containing kanamycin and 100 ng/mL anhydrotetracycline (ATc; IBA Life sciences) for the induction of the S. thermophilus CRISPR1-Cas9 system. Single colonies were picked and screened for mutations in the gene of interest by PCR and sequencing. After verification, the CRISPR1-Cas9 integrative plasmid was removed by exchanging it with pTdTomato-L5, which integrates at the same site.

### RNA isolation and quantitative RT-PCR.

RNA was isolated from bacterial cultures grown to an optical density at 600 nm (OD_600_) of 1. Bacteria were lysed by bead beating in the presence of buffer RA-1 (NucleoSpin RNA isolation kit; Mackery-Nagel) and β-mercaptoethanol (Sigma). RNA isolation and purification were continued according to the protocol supplied by the manufacturer (NucleoSpin RNA isolation kit; Mackery-Nagel). Samples were eluted in water with Ribolock (Thermoscientific), and an additional DNase treatment was performed with DNase I (Thermoscientific) according to the protocol supplied by the manufacturer. cDNA was synthesized from 400 ng RNA with the RevertAid first-strand cDNA synthesis kit (Thermoscientific). Prior to the PCRs, the cDNA was diluted 1:10 in RNase-free water. Reactions were set up using iTaq universal SYBR green supermix (Bio-Rad). Quantitative reverse transcription-PCR (RT-PCR) was performed with the QuantStudio 3 real-time PCR system (ThermoFisher). At the end of amplification, PCR product specificity was verified by melt curve analysis and agarose gel electrophoresis. The threshold cycle (*C_T_*) values were normalized with the *C_T_* of SigA.

### Protein secretion.

M. marinum cultures were grown until mid-logarithmic phase (OD_600_ of 1 to 1.4), before they were washed with 7H9 liquid medium containing 0.2% dextrose and 0.05% Tween 80 to clear the cultures of bovine serum albumin (BSA). Cultures were set at an OD_600_ 0.4 to 0.5 and grown for 16 h. Cells were pelleted (10 min at 3,000 × *g*), washed, and resuspended in phosphate-buffered saline (PBS) before lysing by bead beating, and a 1/4 volume of 5× SDS loading buffer (312.5 mM Tris-HCl [pH 6.8], 10% SDS, 30% glycerol, 500 mM dithiothreitol, and 0.2% bromophenol blue) was added, yielding the pellet fraction. Culture medium was passed through an 0.22-μm filter, and proteins were precipitated with trichloroacetic acid (TCA). TCA-precipitated pellets were washed with acetone and resuspended in 1× SDS loading buffer, yielding the supernatant fraction. Alternatively, a fraction of pelleted bacteria was incubated with 0.5% Genapol X-080 in PBS for 30 min with rotation at room temperature to extract cell surface-associated proteins. Genapol-treated cells were pelleted, washed, and resuspended in PBS before lysis by bead beating and addition of a 1/4 volume of 5× SDS buffer, yielding the Genapol pellet fraction. A 1/4 volume of 5× SDS buffer was also added to Genapol supernatants, yielding the Genapol supernatant fraction. All fractions were boiled for 10 min at 95°C before loading on SDS-PAGE gels.

### Fractionation and Strep-tagged protein purification.

Mycobacterial cultures were grown to an OD_600_ of 1. Cells were harvested and washed with PBS before resuspension in PBS with 10% glycerol to a concentration of 50 to 100 OD/mL. Cells were lysed by passage through a One-Shot cell disrupter (Constant Systems) at 0.83 kbar. Unbroken cells were pelleted by centrifugation at 15,000 × *g* for 10 min at 4°C. The obtained supernatant was subjected to ultracentrifugation at 100,000 × *g* for 1 h at 4°C, yielding the cell envelope pellet and soluble fractions. Samples were boiled in SDS buffer for 10 min at 95°C before loading on SDS-PAGE gels, as explained above. Alternatively, soluble fractions were then separated under native conditions on a 4 to 16% NativePage Novex BisTris protein gel (Life Technologies) and transferred to a polyvinylidene difluoride (PVDF) membrane before immunostaining.

For the purification of intracellular Strep-tagged proteins, cells were harvested and lysed in buffer B (PBS with 10% glycerol [pH 8]). Soluble fractions were prepared and incubated with StrepTactin resin (IBA Lifesciences) for 30 min with rotation at 4°C. Beads were then washed with buffer B and proteins were eluted using buffer B supplemented with 10 mM desthiobiotin (IBA Lifesciences). Samples were taken at each step of the purification and loaded on SDS-PAGE gels to check the efficiency of affinity purification. Elution fractions were also separated under native conditions to ascertain the stability of the complexes after affinity purification.

### Protein detection and sera.

SDS-PAGE and NativePage gels were stained with Brilliant Blue G-250 or R-250 (CBB; Bio-Rad), respectively. Alternatively, proteins were transferred to nitrocellulose (SDS-PAGE) or PVDF (BN-PAGE) membranes. Proteins were visualized using standard immunodetection techniques. Primary antibodies used in this study were anti-GroEL2 (CS44; Colorado State University), anti-PE_PGRS antibody (7C4.1F7) (77), anti-EsxA (Hyb76-8) (78), polyclonal anti-FtsH (79), polyclonal anti-PPE68 (9), polyclonal anti-EspE (51, 52), and polyclonal anti-Strep (Novusbio).

### Mass spectrometry.

To identify the interaction partners of PPE68, elution fractions of the Strep-tagged protein purifications were analyzed by LC-MS/MS, essentially as described before (80). In short, SDS loading buffer was added to elution samples of Strep-tagged protein purification and boiled for 10 min at 95°C before loading on SDS-PAGE gels. SDS-PAGE gels were stained by CBB, and total protein lanes were excised, washed, and processed for in-gel digestion. Peptides were eluted from the gel pieces and analyzed by the Ultimate 3000 nanoLC-MS/MS system (Dionex LC-Packings, Amsterdam, The Netherlands). Proteins were identified from the resulting LC-MS/MS spectra by searching against the Uniprot M. marinum complete proteome (ATCC BAA-535M).

### Hemolysis assay.

M. marinum strains were grown in 7H9 medium supplemented with ADC and 0.05% Tween 80 until mid-logarithmic phase. All strains were washed with PBS and diluted in Dulbecco's modified Eagle medium (DMEM) without phenol red (Gibco, Life Technologies). Numbers of bacteria were quantified by absorbance measurement at OD_600_ with an estimation of 2.5 × 10^8^ bacteria in 1 mL of a 1.0 OD_600_ suspension. Defibrinated sheep erythrocytes (Oxoid-Thermo Fisher, The Netherlands) were washed and diluted in DMEM. A total of 4.15 × 10^7^ erythrocytes and 1.04 × 10^8^ bacteria (1:2.5 ratio) were added for one reaction mixture of 100 μL in a round-bottom 96-well plate, gently centrifuged for 5 min, and incubated at 32°C with 5% CO_2_. After 3 h of incubation, cells were resuspended and centrifuged. Supernatants were transferred to a flat-bottom 96-well plate and measured at an absorbance of 405 nm to quantify hemoglobin release.
