# Supplementary figures and images for: The ESX-1 Substrate PPE68 Has a Key Function in ESX-1-Mediated Secretion in Mycobacterium marinum
Source: mBio. 2022 Nov 21;13(6):e02819-22. doi: 10.1128/mbio.02819-22 (PMC9765416; doi:10.1128/mbio.02819-22)

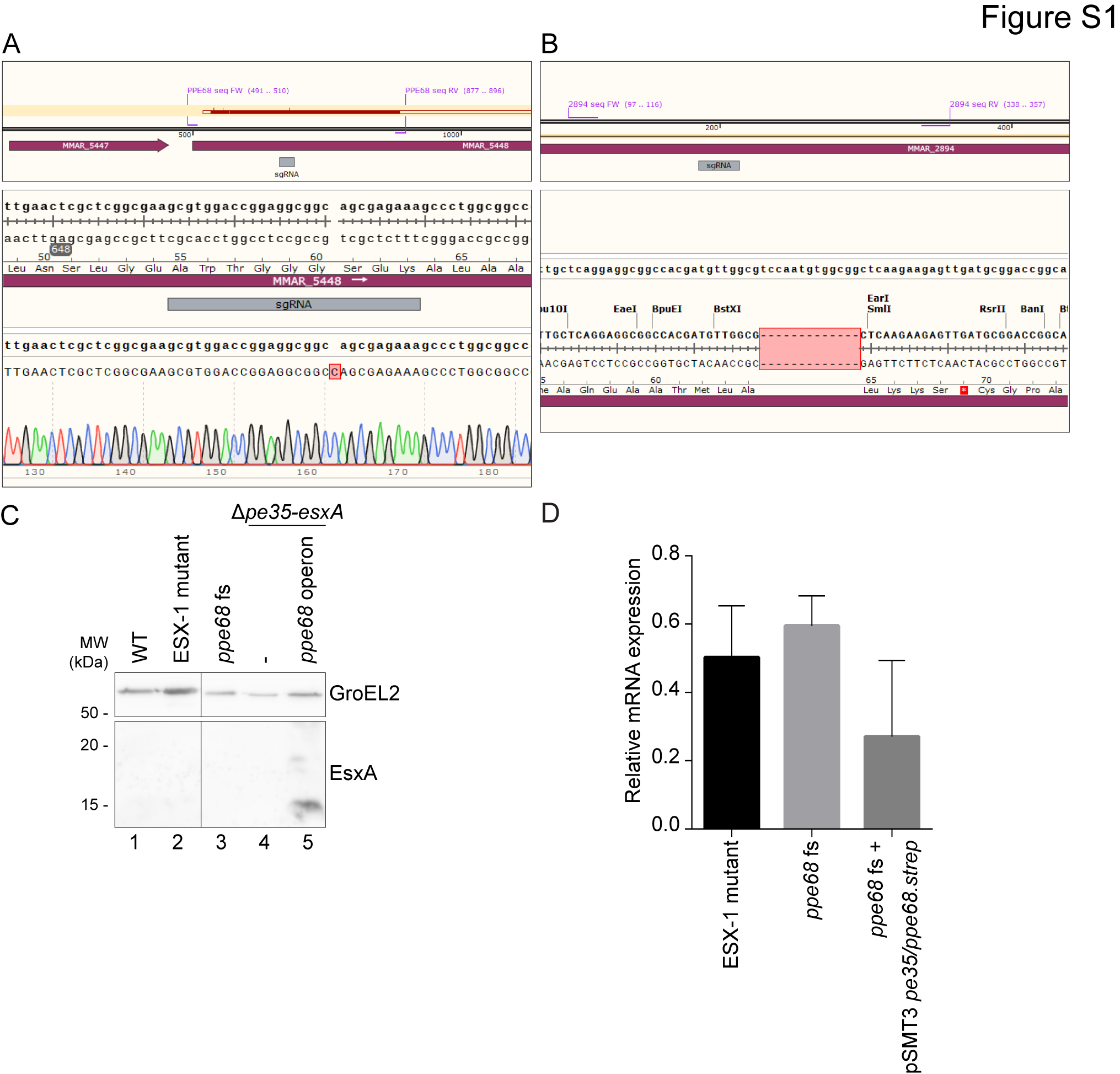

Supplement: FIG S1 [file mbio.02819-22-s0001.tif]

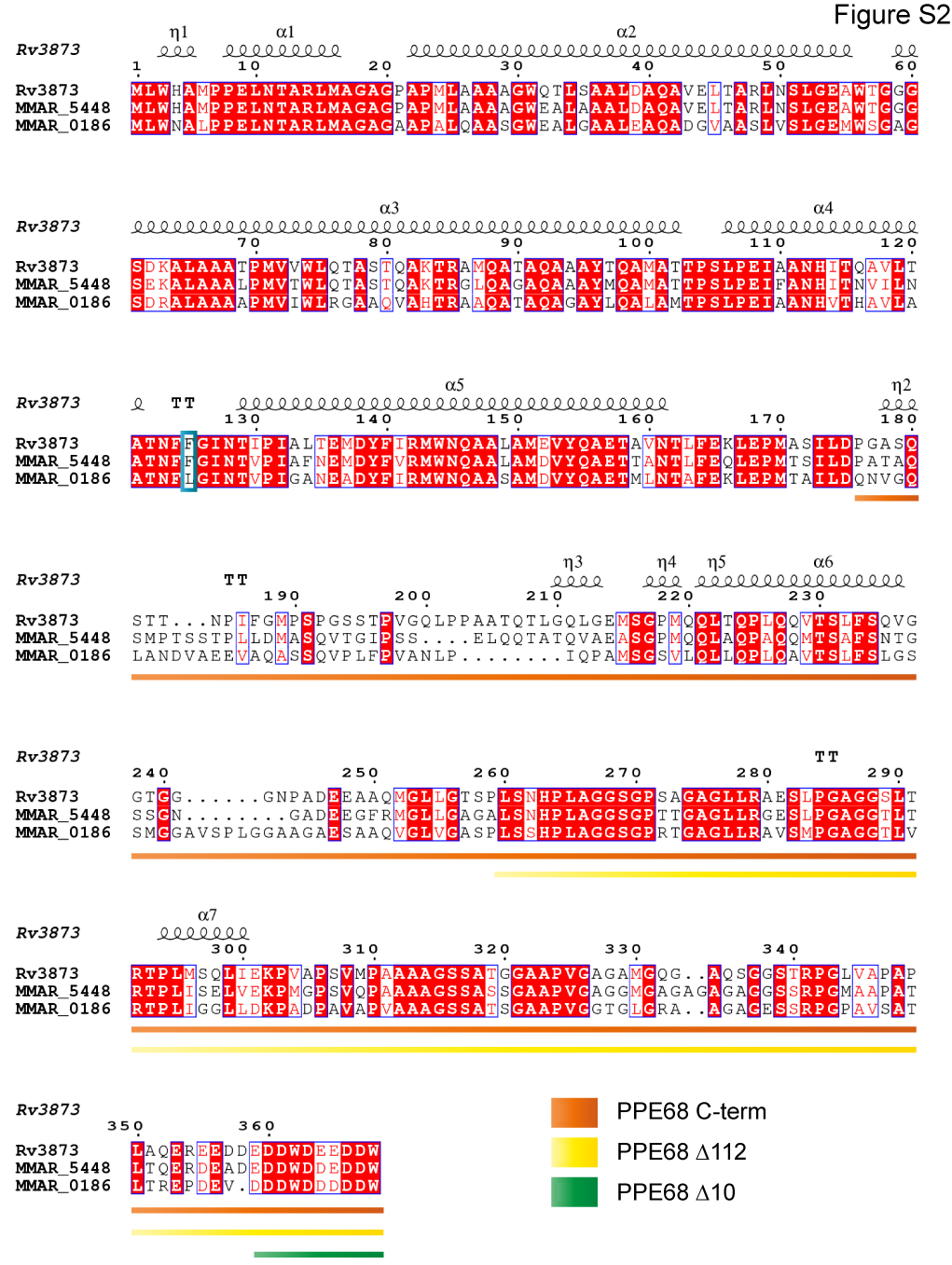

Supplement: FIG S2 [file mbio.02819-22-s0002.tif]

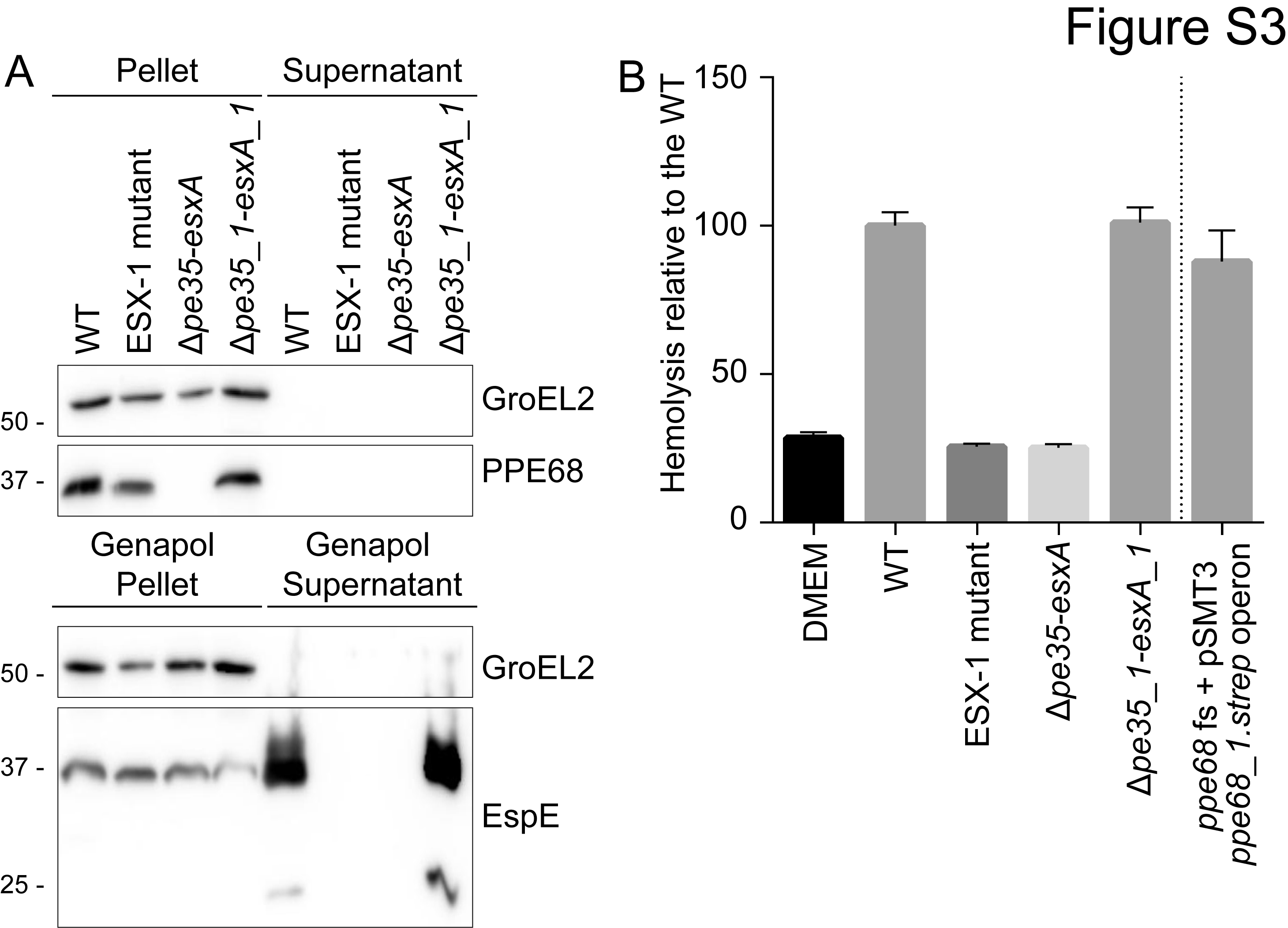

Supplement: FIG S3 [file mbio.02819-22-s0003.tif]

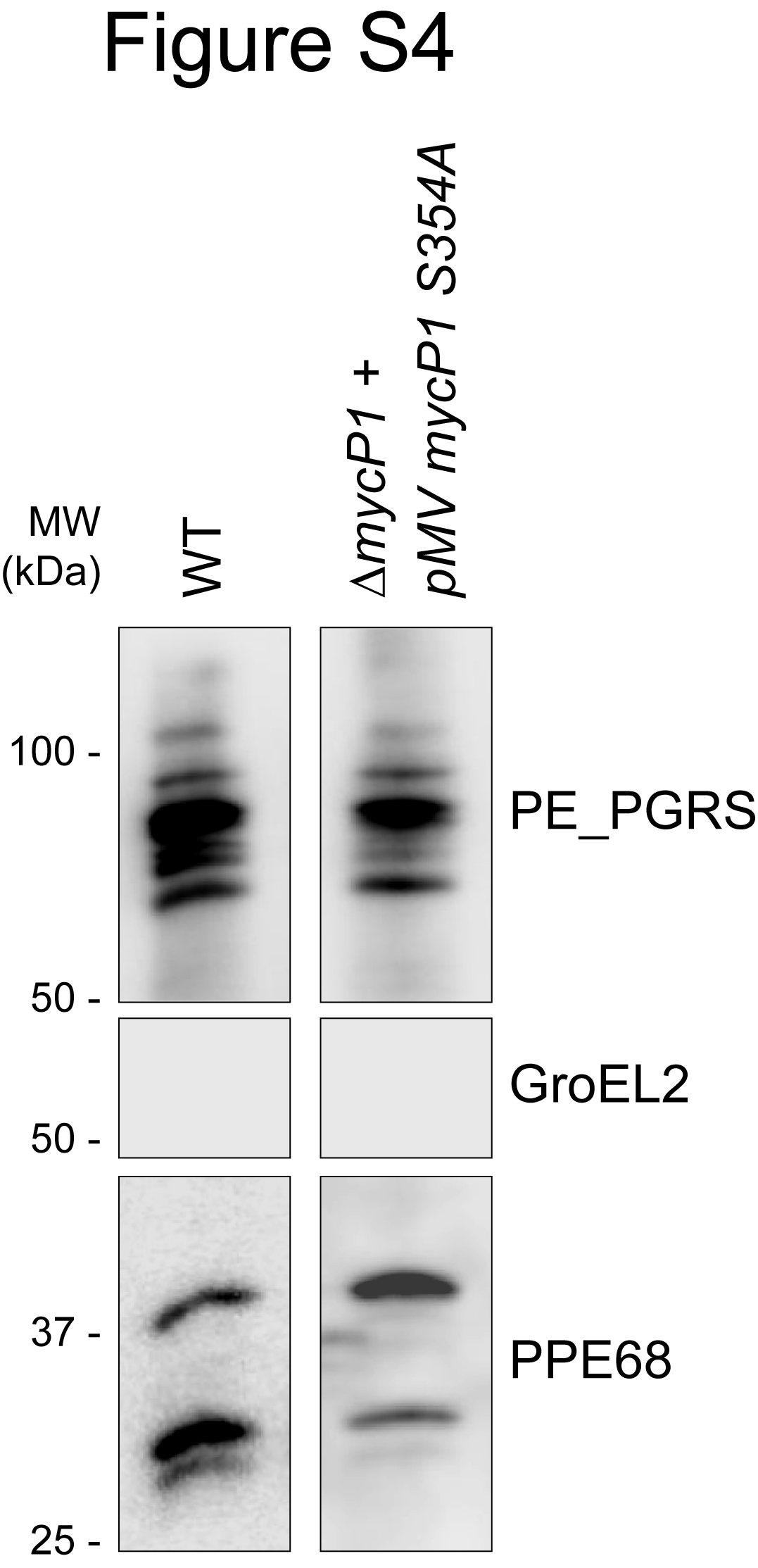

Supplement: FIG S4 [file mbio.02819-22-s0004.tif]

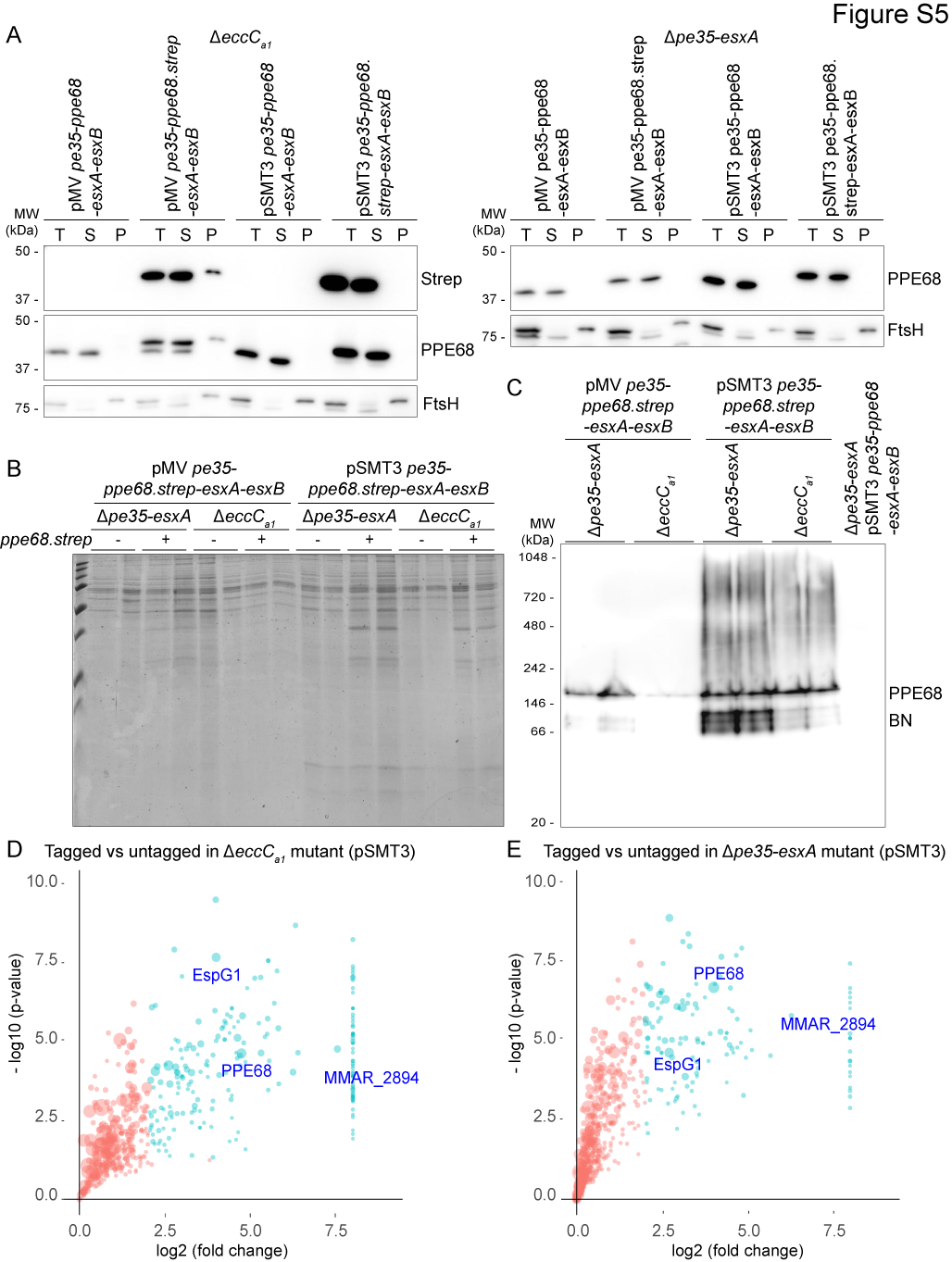

Supplement: FIG S5 [file mbio.02819-22-s0005.tif]

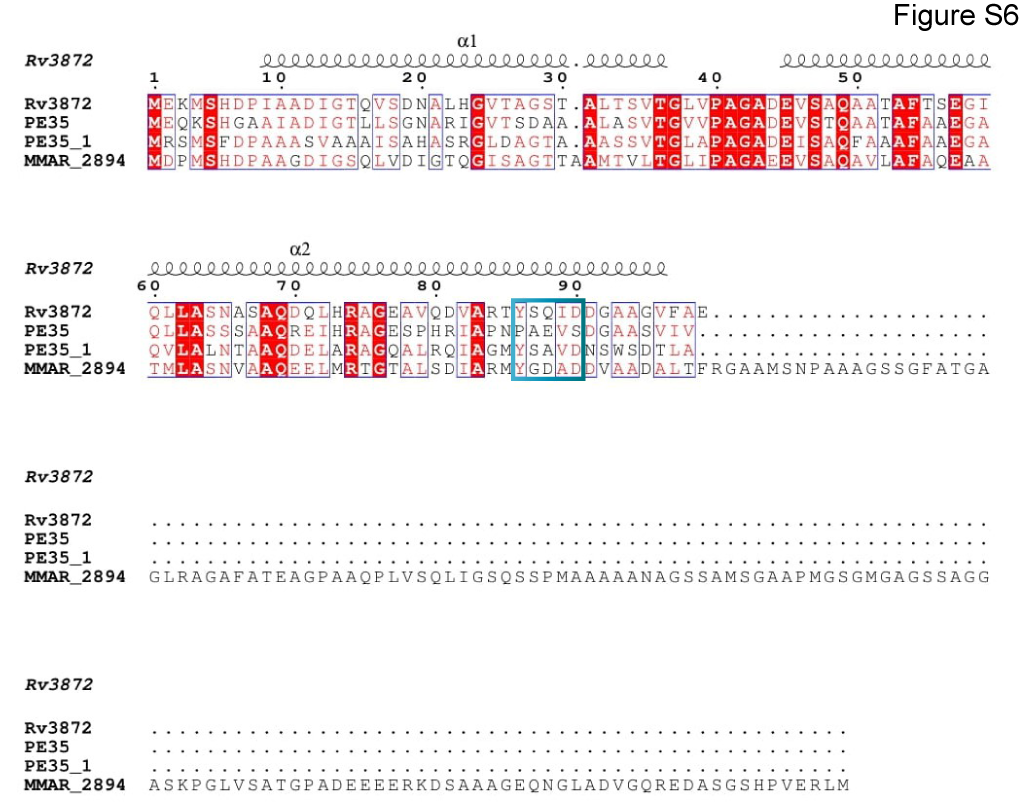

Supplement: FIG S6 [file mbio.02819-22-s0006.tif]

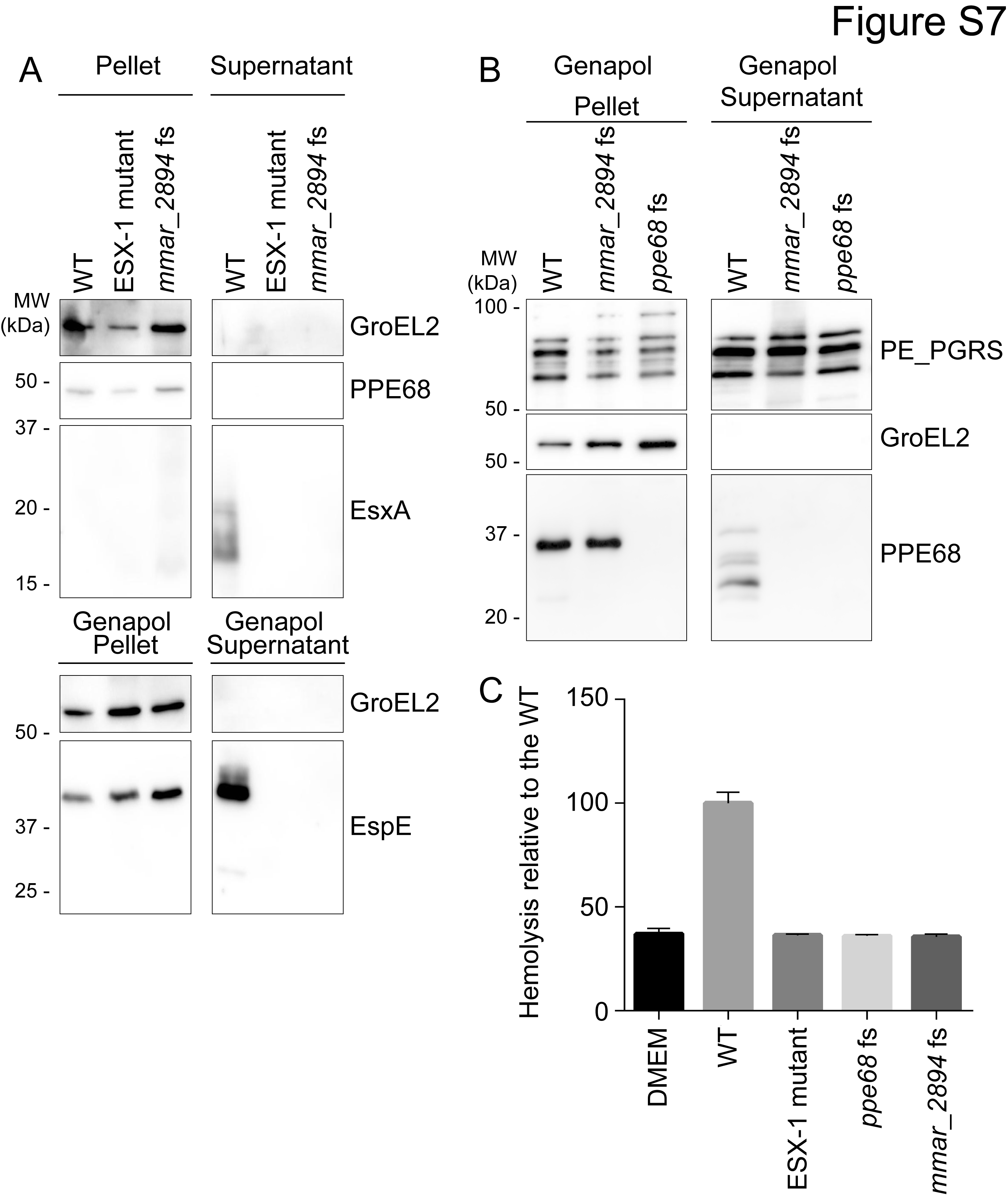

Supplement: FIG S7 [file mbio.02819-22-s0007.tif]
