# Supplementary material for: The ESX-1 Substrate PPE68 Has a Key Function in ESX-1-Mediated Secretion in Mycobacterium marinum
Source: mBio. 2022 Nov 21;13(6):e02819-22. doi: 10.1128/mbio.02819-22 (PMC9765416; doi:10.1128/mbio.02819-22)
Supplement: TABLE S1 [file mbio.02819-22-s0008.docx]

**Table S1.** Significant hits of PPE68 interactors as determined by protein purification and mass spectrometry analysis. Only proteins with an average normalized spectral count >5 in the tagged samples, a log2 fold change > 2 and a -log10 p-value of > 1.3 between tagged and untagged samples are shown.

| **Δ*EccCa1*::pMV *pe35-esxA*** | | | | | **Δ*pe35-esxA*::pMV *pe35-esxA*** | | | | |
| --- | --- | --- | --- | --- | --- | --- | --- | --- | --- |
| **Protein** | Average raw counts tagged | Average raw counts untagged | log2 fold change | -log10 P-value | **Protein** | Average raw counts tagged | Average raw counts untagged | log2 fold change | -log10 P-value |
| **EspG_1_** | 149.00 | 20.00 | 2.90 | 3.62 | **EspG_1_** | 150.50 | 17.50 | 3.10 | 5.99 |
| **PPE68** | 81.50 | 7.00 | 3.54 | 4.80 | **PPE68** | 115.00 | 8.50 | 3.76 | 3.58 |
| **MMAR_2894** | 15.50 | 0.00 | 8.00 | 2.59 | **MMAR_2894** | 36.00 | 0.00 | 8.00 | 5.79 |
|  |  |  |  |  | **NrdE** | 22.50 | 5.00 | 2.17 | 1.44 |
|  |  |  |  |  | **HemD** | 11.00 | 0.00 | 8.00 | 1.57 |
|  |  |  |  |  | **FadH** | 11.00 | 1.00 | 3.46 | 3.07 |
|  |  |  |  |  | **HupB** | 10.50 | 0.50 | 4.39 | 2.25 |
|  |  |  |  |  | **FtsE** | 9.00 | 1.50 | 2.58 | 2.02 |
|  |  |  |  |  | **MMAR_2279** | 8.50 | 0.00 | 8.00 | 1.34 |
|  |  |  |  |  | **FabD** | 8.00 | 0.50 | 4.00 | 1.59 |
|  |  |  |  |  | **RplW** | 8.00 | 1.50 | 2.42 | 2.11 |
|  |  |  |  |  | **RhlE** | 7.50 | 1.00 | 2.91 | 1.99 |
|  |  |  |  |  | **MMAR_5479** | 7.00 | 1.00 | 2.81 | 1.62 |
|  |  |  |  |  | **RpsS** | 6.50 | 0.00 | 8.00 | 1.59 |
|  |  |  |  |  | **PlsC** | 6.00 | 0.00 | 8.00 | 1.30 |
|  |  |  |  |  | **MMAR_1001** | 6.00 | 0.00 | 8.00 | 1.30 |
|  |  |  |  |  | **AhpC** | 5.50 | 0.00 | 8.00 | 2.70 |
|  |  |  |  |  | **RpmF** | 5.50 | 1.00 | 2.46 | 2.04 |
|  |  |  |  |  | **AsnB** | 5.50 | 1.00 | 2.46 | 1.54 |
|  |  |  |  |  | **ProB** | 5.50 | 1.00 | 2.46 | 1.44 |
|  |  |  |  |  | **ChoD_1** | 5.50 | 0.00 | 8.00 | 1.84 |
