# Supplementary material for: The ESX-1 Substrate PPE68 Has a Key Function in ESX-1-Mediated Secretion in Mycobacterium marinum
Source: mBio. 2022 Nov 21;13(6):e02819-22. doi: 10.1128/mbio.02819-22 (PMC9765416; doi:10.1128/mbio.02819-22)
Supplement: TABLE S2 [file mbio.02819-22-s0009.docx]

**Table S2.** Strains and plasmids used in this study.

| Strain/plasmid | Characteristics | | | Origin | | Reference |
| --- | --- | --- | --- | --- | --- | --- |
| *M. marinum* M | Wild-type | | |  | | (Abdallah et al., 2006) |
| *M. marinum* Δ*eccC_b1_* | ESX-1 complex mutant | | | *M. marinum* M | | (Abdallah et al., 2009) |
| *M. marinum* Δ*eccC_a1_* | ESX-1 complex mutant | | | *M. marinum* M | | This study |
| *M. marinum* Δ*espG_1_* | ESX-1 chaperone mutant | | | *M. marinum* M | | (Phan et al., 2017) |
| *M. tuberculosis* mc^2^6020 | Δ*LysA* Δ*panCD* | | | *M. tuberculosis* H37Rv | | (Sambandamurthy et al., 2005) |
| *M. marinum* *ppe68* fs | CP000854.1 g6.590.143_6.590.144insC | | | *M. marinum* M | | This study |
| *M. marinum*  *mmar_2894* fs | CP000854.1 g3.498.899_3.498.911del | | | *M. marinum* M | | This study |
| *M. marinum* *pecABC* fs |  | | | *M. marinum* M | | Manuscript in progress  (Meijers *et al*.) |
| *M. marinum* Δ*mmar_5447-50* |  | | | *M. marinum* M | | This study |
| *M. marinum* Δ*mmar_0185-88* |  | | | *M. marinum* M | | This study |
| pMV::*esxB/esxA* | pMV361 backbone, hsp60 promoter, hygR, integrative | | | WT *M. marinum* M, *mmar_5449-50* | | This study |
| pMV::*mmar_5447/ppe68* | pMV361 backbone hsp60 promoter, hygR, integrative | | | WT *M. marinum* M, *mmar_5447-48* | | This study |
| pMV::*mmar_5447/ppe68/*  *esxB/esxA* | pMV361 backbone hsp60 promoter, hygR, integrative | | | WT *M. marinum* M, *mmar_5447-50* | | This study |
| pSMT3::*mmar_5447/ppe68/*  *esxB/esxA* | pSMT3 backbone, hsp60 promoter, hygR, multicopy | | | WT *M. marinum* M, *mmar_5447-50* | | This study |
| pSMT3::*pe35/ppe68/esxB/*  *esxA* | pSMT3 backbone, hsp60 promoter, hygR, multicopy | | | WT *M. tuberculosis H37Rv, rv3872-75* | | This study |
| pMV::*mmar_5447/ppe68.*  *strep/esxB/esxA* | pMV361 backbone hsp60 promoter, hygR, integrative | | | WT *M. marinum* M, *mmar_5447-50* | | This study |
| pSMT3::*mmar_5447/ppe68.*  *strep* | pSMT3 backbone, hsp60 promoter, hygR, multicopy | | | WT *M. marinum* M, *mmar_5447-48* | | This study |
| pSMT3::*mmar_5447/ppe68.*  *strep/esxB/esxA* | pSMT3 backbone, hsp60 promoter, hygR, multicopy | WT *M. marinum* M, *mmar_5447-50* | | | | This study |
| pSMT3::*mmar_5447/ppe68*  *ΔCterm.strep/esxB/esxA* | pSMT3 backbone, hsp60 promoter, hygR, multicopy | WT *M. marinum* M, *mmar_5447-50* | | | | This study |
| pSMT3::*pe35/ppe68_1.strep /esxB_1/esxA_1* | pSMT3 backbone, hsp60 promoter, hygR, multicopy | WT *M. marinum* M, *mmar_0185-88* | | | | This study |
| pSMT3::*mmar_5447/*  *ppe68_1ΔC-term + C-term ppe68.strep /esxB/esxA* | pSMT3 backbone, hsp60 promoter, hygR, multicopy | WT *M. marinum* M, *mmar_0185-86, mmar_5448-50* | | | | This study |
| pSMT3::*mmar_5447/ppe68*  *F125A.strep /esxB/esxA* | pSMT3 backbone, hsp60 promoter, hygR, multicopy | WT *M. marinum* M, *mmar_5447-50* | | | | This study |
| pSMT3::*mmar_5447/ppe68_1ΔC-term L125A + C-term ppe68.strep/esxB/esxA* | pSMT3 backbone, hsp60 promoter, hygR, multicopy | | WT *M. marinum* M, *mmar_0185-86, mmar_5448-50* | | | This study |
| pSMT3::*mmar_5447/ppe68*  *ΔC10.strep/esxB/esxA* | pSMT3 backbone, hsp60 promoter, hygR, multicopy | | | WT *M. marinum* M, *mmar_5447-50* | | This study |
| pSMT3::*mmar_5447/ppe68*  *ΔC112.strep/esxB/esxA* | pSMT3 backbone, hsp60 promoter, hygR, multicopy | | | WT *M. marinum* M, *mmar_5447-50* | | This study |
| pTdTomato-L5 | pMV361 backbone hsp60 promoter, StrepR, integrative | | | |  | Meijers 2020 Addgene 140994 |
| pCRISPRx-Sth1Cas9-L5::sgRNA-*ppe68* | TetR, KanR, integrative | | | | WT *M. marinum* M, *mmar_5448* | This study, based on Meijers 2020 Addgene 140993 |
| pCRISPRx-Sth1Cas9-L5::sgRNA-*mmar_2894* | TetR, KanR, integrative | | | | WT *M. marinum* M, *mmar_2894* | This study, based on Meijers 2020 Addgene 140993 |
