# Supplementary material for: The ESX-1 Substrate PPE68 Has a Key Function in ESX-1-Mediated Secretion in Mycobacterium marinum
Source: mBio. 2022 Nov 21;13(6):e02819-22. doi: 10.1128/mbio.02819-22 (PMC9765416; doi:10.1128/mbio.02819-22)
Supplement: TABLE S3 [file mbio.02819-22-s0010.docx]

**Table S3.** Primers used in this study.

| Oligonucleotide | Sequence | Use |
| --- | --- | --- |
| EsxB_XmnI_FW | TTTGAAAGAATTCCATATGGCAGAGATGAAGACCGATGCC | Cloning *esxB*/*esxA* into pMV |
| EsxA_HindIII_RV | TTTAAGCTTTTAAGCAAACATCCCCGTGACGTT | Cloning *esxB*/*esxA* into pMV |
| Pe35_XmnI_FW | TTTGAAAGAATTCATGGAACAAAAGTCACACGGCG | Cloning *pe35*/*ppe68* into pMV |
| Ppe68_HindIII_RV | TTTAAGCTTTCACCAGTCGTCCTCGTCATCCCA | Cloning *pe35*/*ppe68* into pMV |
| Pe35_NheI_FW | TTTGCTAGCATGGAACAAAAGTCACACGGCG | Cloning *pe35*/*ppe68*/*esxB*/*esxA* into pSMT3 |
| EsxA_BamHI_RV | TTTGGATCCTTAAGCAAACATCCCCGTGACGTT | Cloning *pe35*/*ppe68*/*esxB*/*esxA* into pSMT3 |
| PPE68.strep_BamHI_RV | TTTGGATCCTCACTTCTCGAACTGCGGGTGG | Cloning *pe35*/*ppe68* into pSMT3 |
| PPE68_strep_RV | AACTGCGGGTGGCTCCAAGCGCTCCAGTCGTCCTCGTCATCCCA | Cloning Strep-tag sequence into *ppe68* |
| Ppe68_EsxB_strep_FW | GAGCCACCCGCAGTTCGAGAAGTGAGCGTCGTCAACACGAAC | Cloning Strep-tag sequence into *ppe68* |
| Pe35mtb_NheI_FW | TTTGCTAGCATGGAAAAAATGTCACATGATCCGATCG | Cloning *rv3872-75* into pSMT3 |
| EsxAmtb_BamHI_RV | TTTGGATCCCTATGCGAACATCCCAGTGACG | Cloning *rv3872-75* into pSMT3 |
| NheI_PE35_1_FW | GAATCACGCTAGCatgCGATCC | Cloning *mmar_0185-88* into pSMT3 |
| BamHI_EsxA_1_RV | ATCCATGGATCCTAGCCGAACA | Cloning *mmar_0185-88* into pSMT3 |
| PPE68dC_strep_RV | cgggtggctccaagcgctCTGTGCAGTGGCGGGAT | Cloning *ppe68* with a C-terminal deletion after 525 bp (175 amino acids) |
| PPE68dC_strep_FW | ATCCCGCCACTGCACAGagcgcttggagccacccg | Cloning *ppe68* with a C-terminal deletion after 525 bp (175 amino acids) |
| PPE68_1_strep_RV | aactgcgggtggctccaagcgctCCAGTCGTCGTCGTCATCCC | Cloning Strep-tag sequence into *ppe68_1* |
| PPE68_1_strep_FW | gagccacccgcagttcgagaagTGAGAGTCGTTGCTAGGAAA | Cloning Strep-tag sequence into *ppe68_1* |
| PPE68tub_strep_RV | aactgcgggtggctccaagcgctCCAGTCGTCCTCTTCGTCCC | Cloning Strep-tag sequence into *rv3875* |
| PPE68tub_strep_FW | gagccacccgcagttcgagaagTGAGCTCCCGTAATGACAACA | Cloning Strep-tag sequence into *rv3875* |
| PPE68 cut FW | GGGA GCGTGGACCGGAGGCGGCAG | sgRNA for generation of frameshift mutant |
| PPE68 cut RV | AAAC CTGCCGCCTCCGGTCCACGC | sgRNA for generation of frameshift mutant |
| 2894_FW | AAAC AGCCGCCACATTGGACGCCAAC | sgRNA for generation of frameshift mutant |
| 2894_RV | GGGA GTTGGCGTCCAATGTGGCGGCT | sgRNA for generation of frameshift mutant |
| MMAR_5447-50 Left Flank Fw | TTTTTTTTCACAAAGTGGCCTTCGTCAGTGAGTTTCC | Generation of knockout strains |
| MMAR_5447-50 Left Flank Rev | TTTTTTTTCACTTCGTGAGCGTTGCCGCTCAATAGTG | Generation of knockout strains |
| MMAR_5447-50 RF Fw2 | TTTTTTTTCACAGAGTGAGGCCGGTCAGGCAATGTCC | Generation of knockout strains |
| MMAR_5447-50 RF Rev2 | TTTTTTTTCACCTTGTGCTGCGGTGGCTCTGCTGAGG | Generation of knockout strains |
| MMAR_0185-88 KO LF | TTTTTTTTCCATAAATTGGTGACGCGGACAGCGTTAATA | Generation of knockout strains |
| MMAR_0185-88 KO LR | TTTTTTTTCCATTTCTTGGTGCACAACTCCTCTGCACTA | Generation of knockout strains |
| MMAR_0185-88 KO RF | TTTTTTTTCCATAGATTGGAGGCCGGTCAGGCAATGTCC | Generation of knockout strains |
| MMAR_0185-88 KO RR | TTTTTTTTCCATCTTTTGGCGGTCGAGTGCAGCCACCAA | Generation of knockout strains |
| EccC1 KO LF | TTTTTTTTCACAAAGTGGCGATGCTGGCGGACTATCA | Generation of knockout strains |
| EccC1 KO LR | TTTTTTTTCACTTCGTGGTGCCGACGATGGACAGAAG | Generation of knockout strains |
| EccC1 KO RF | TTTTTTTTCACAGAGTGTTGCGTACCACCAGCTCTCA | Generation of knockout strains |
| EccC1 KO RR | TTTTTTTTCACCTTGTGGGTGTGCATGTGGCTGTAAG | Generation of knockout strains |
| SigA-FW | GAAAAACCACCTGCTGGAAG | (Phan et al., 2017) |
| SigA-RV | CGCGTAGGTGGAGAACTTGT | (Phan et al., 2017) |
| EsxA3 qPCR F | GCATCCAGCGCAATTCAGGG | (Phan et al., 2017) |
| EsxA3 qPCR R | GCGAGTTGTTGAGCTCCTGC | (Phan et al., 2017) |
